# Dynamic coordination of two-metal-ions orchestrates λ-exonuclease catalysis

**DOI:** 10.1038/s41467-018-06750-9

**Published:** 2018-10-23

**Authors:** Wonseok Hwang, Jungmin Yoo, Yuno Lee, Suyeon Park, Phuong Lien Hoang, HyeokJin Cho, Jeongmin Yu, Thi Minh Hoa Vo, Minsang Shin, Mi Sun Jin, Daeho Park, Changbong Hyeon, Gwangrog Lee

**Affiliations:** 1 Korea Institute for Advanced Study, Seoul, 02455 Republic of Korea; 2 School of Life Sciences, Gwangju Institute of Science and Technology, Gwangju, 61005 Republic of Korea; 3 Department of Microbiology, School of Medicine, Kyungpook National University, 680 Gukchaebosang-Ro, Jung-gu, Daegu 41944 Republic of Korea; 4 Present Address: Clova AI Research, NAVER Corp, Seongnam, 13561 Republic of Korea; 5 Present Address: Korea Research Institute of Chemical Technology, Daejeon, 34114 Republic of Korea

## Abstract

Metal ions at the active site of an enzyme act as cofactors, and their dynamic fluctuations can potentially influence enzyme activity. Here, we use λ-exonuclease as a model enzyme with two Mg^2+^ binding sites and probe activity at various concentrations of magnesium by single-molecule-FRET. We find that while Mg_A_^2+^ and Mg_B_^2+^ have similar binding constants, the dissociation rate of Mg_A_^2+^ is two order of magnitude lower than that of Mg_B_^2+^ due to a kinetic-barrier-difference. At physiological Mg^2+^ concentration, the Mg_B_^2+^ ion near the 5’-terminal side of the scissile phosphate dissociates each-round of degradation, facilitating a series of DNA cleavages via fast product-release concomitant with enzyme-translocation. At a low magnesium concentration, occasional dissociation and slow re-coordination of Mg_A_^2+^ result in pauses during processive degradation. Our study highlights the importance of metal-ion-coordination dynamics in correlation with the enzymatic reaction-steps, and offers insights into the origin of dynamic heterogeneity in enzymatic catalysis.

## Abstract (web summary)

Metal ions at the active site of an enzyme act as cofactors and their dynamic fluctuations might influence enzyme activity. Here authors use single-molecule FRET to study λ-exonuclease and find that metal-ion-coordination is correlated with enzymatic reaction-steps.

## Introduction

Mg^2+^ is an abundant divalent cation in cells that is required by many enzymes^1–3^ that involve breaking and forming phosphodiester bonds during nucleic acid metabolism. Many studies have been devoted to better understanding various metal-ion binding modes^4^, phosphodiester-cleavage reactions, and the binding free energies of different ligand coordination systems^5,6^. Among diverse metal-ion chemistries, the two-metal-ion coordination is frequently used by nucleases, polymerases, integrases, ATPases, and topoisomerases. Furthermore, several time-resolved X-ray crystallographic analyses have revealed dynamic changes in the metal-ion coordination at the active site during DNA cleavage^7,8^ and DNA synthesis^9,10^.

Most theoretical studies however have been limited to the cleavage steps^11–14^ and rarely investigated the stability or residence time of metal-ion cofactors at the active site. In principle, metal ions coordinated to the active site of an enzyme have finite lifetimes. If the coordinated lifetime is shorter than or comparable to the time scale associated with the enzyme cycle, then the dynamics of metal ions could affect the rate and dynamics of enzymatic turnover, and in turn cause dynamic disorder in the catalytic cycle of the enzyme^15^. Although some results have suggested that two Mg^2+^ ions in nucleases have separate catalytic roles^16–18^ (e.g., Mg_A_^2+^ primes the activation of the nucleophile, while Mg_B_^2+^ stabilizes the transition state and accelerates product formation), the details of their roles during the full enzymatic cycle are not fully understood. In this study, we use λ-exonuclease as a model system to investigate the microscopic underpinnings of how the two catalytic metal ions promote catalysis and address how their dynamics affect overall enzymatic activity. We adopt single-molecule fluorescence resonance energy transfer (FRET) as an experimental tool to directly probe the time-resolved dynamics of exonuclease translocation along a DNA strand. We perform kinetic modeling of Mg^2+^-dependent degradation and molecular dynamics (MD) simulations to correlate the metal-ion chemistry and enzyme activity (including cleavage and translocation) and to infer a molecular mechanism that can best explain the diverse patterns observed in the FRET time trajectories. We find that the two metal ions in the active site (Mg_A_^2+^ and Mg_B_^2+^) have similar binding constants, but asymmetric thermodynamic stabilities during the enzymatic reaction.

## RESULTS

### Single molecule fluorescence assay for λ-exonuclease

**Fig. 1:**
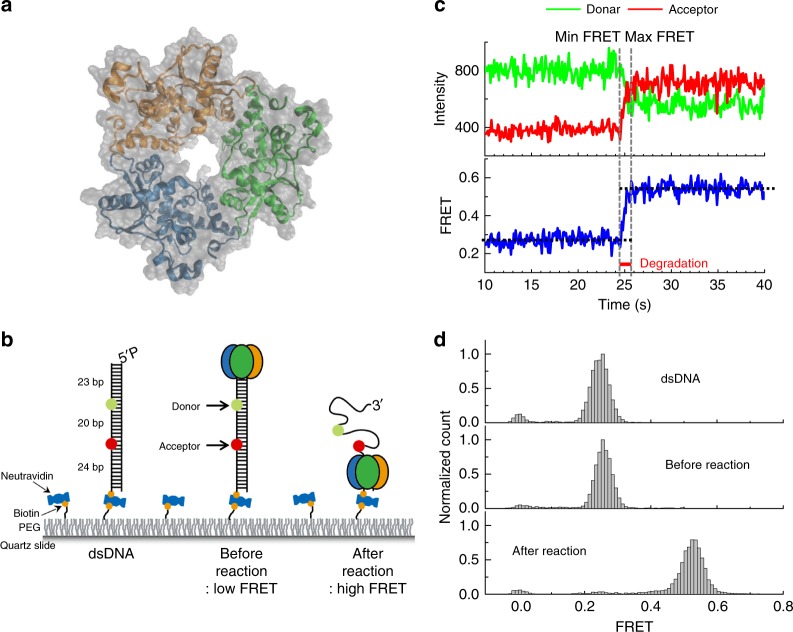
Real-time measurements of processive degradation by λ-exonuclease. **a** The crystal structure of λ-exonuclease (PDB entry 1AVQ). **b** Experimental layout, depicting the DNA, protein binding to DNA, and processive degradation. **c** Schematic showing how the degradation time is measured using FRET signal. **d** Single-molecule FRET histogram obtained as in **b** (top: dsDNA only; middle: before the degradation in the absence of Mg^2+^; and bottom: after the degradation in the presence of Mg^2+^)

λ-exonuclease, which forms a homotrimeric ring structure wrapped around the linear DNA duplex^19,20^, degrades one strand of the DNA in the 5′-to-3′ direction, producing a 3′ single-stranded (ss) overhang tail (Fig. 1)^21–23^. The resulting product serves as a DNA intermediate, essential for homologous recombination in the bacteriophage λ system^24^. λ-exonuclease translocation along DNA is driven by the chemical free energy released by the hydrolysis of phosphodiester bonds^25^ and is a highly processive enzyme^26,27^ (>3,000 nucleotides (nt) per attempt). Mg^2+^, an essential cofactor for the nuclease activity of this enzyme^28^, establishes catalytic coordination at the active site^20^. The co-crystal structure of λ-exonuclease revealed that the enzyme utilizes two Mg^2+^ ions separated by 4.0 Å, both of which prefer an octahedral geometry for catalysis, typical of a classical two-metal mechanism^20^.

To monitor the λ-exonuclease activity in real time at the single-molecule level, we designed a blunt-ended double stranded (ds)DNA substrate with a phosphate group on the 5′ end of the hydrolyzed strand (termed the 5′ strand) and a hydroxyl group on the 3′ end of the non-hydrolyzed strand (termed the 3′ strand). The FRET donor (Cy3) and acceptor (Cy5) were covalently attached to the 23^rd^ and 43^rd^ nucleotides of the duplex, respectively (Fig. 1b). The DNA substrate was immobilized on a polymer-coated quartz surface via biotin-streptavidin interactions (Fig. 1b). This experimental setup, used in our previous study^21,25^, allowed us to monitor the processive phase of degradation in real time.

When a reaction buffer containing Mg^2+^ and λ-exonuclease was applied to DNA molecules immobilized on the surface via a flow delivery system^29^, degradation started from the 5′ strand, with degradation-directed translocation of λ-exonuclease along the 3′ strand (Fig. 1b). The enzymatic degradation converts the rigid dsDNA substrate into flexible ssDNA, which on average shortens the distance between the two fluorophores, producing an increase in FRET (Fig. 1c, d)^21^.

As expected, upon adding Mg^2+^, we observed a gradual increase in the FRET signal (blue trace), caused by a decrease in donor intensity and an increase in accepter intensity (green and red traces in Fig. 1c). The major peak of the FRET efficiency histogram shifted from 0.26 to 0.53 upon cleavage (Fig. 1d). Such FRET changes were not observed in the absence of Mg^2+^ (Fig. 1d, middle panel) and thus can be attributed to λ-exonuclease activity-elicited degradation of DNA^21^. The monotonic increases in FRET without pauses indicate that λ-exonuclease is processive at physiologically relevant Mg^2+^ concentrations (>3 mM)^21^. We characterized the degradation time of the 20-nt-DNA strand between the two FRET dyes by measuring the time period over which FRET increased from the minimum to the maximum values (vertical dotted lines in Fig. 1c). The degradation rates were defined by the number of nucleotides (20 nt) divided by the degradation time

### Mg^2+^-dependent degradation of DNA by λ-exonuclease

**Fig. 2:**
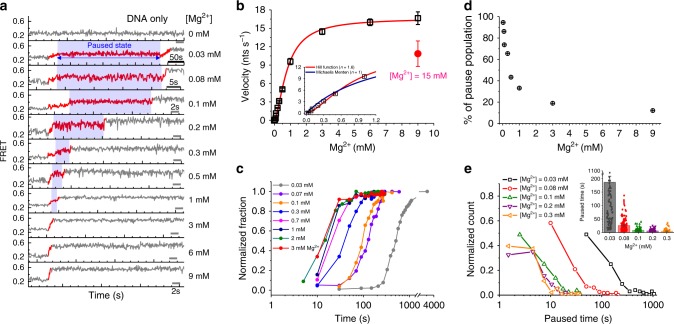
Mg^2+^-dependent degradation of DNA by λ-exonuclease. **a** Representative traces showing that degradation reaction slows down at lower Mg^2+^ concentrations (0 mM, 0.03 mM, 0.08 mM, 0.1 mM, 0.2 mM, 0.3 mM, 0.5 mM, 1 mM, 3 mM, 6 mM, and 9 mM Mg^2+^: top to bottom) at the fixed 16 nM λ-exonuclease (trimer concentration unless otherwise stated). **b** The degradation rate (velocity) versus Mg^2+^ concentrations following a Hill fit (red line) with a maximum velocity of 17.2 nt s^−1^, a Km value of 0.885 mM of Mg^2+^, and *n* = 1.6. Inset, blowing up at lower Mg^2+^ concentrations showing fitting to a sigmoidal kinetics (red) versus a Michaelis Menten kinetics (blue). Error bars denote the standard error of the mean (SEM). The velocity at [Mg^2 +^ ] = 15 mM is highlighted in red. **c** Time-dependent fractional growth at various Mg^2+^ concentrations. **d** The proportion of the pause population as a function of the Mg^2+^ concentration. **e** Distribution of pause times with varying concentrations of Mg^2+^

During the reaction, stable metal-ion coordination is prerequisite for sustaining the persistent enzymatic activity, enabling the enzyme to translocate via continuous degradation of DNA. Thus, the dissociation of metal ions from the active site would change the enzymatic activity. To corroborate whether Mg^2+^ remains at the active site during the reaction, we examined single-molecule trajectories obtained from various Mg^2+^ concentrations and measured the degradation period over which FRET increases from the minimum to the maximum values (red lines in Fig. 2a). Surprisingly, the pattern of the FRET time trajectory (Fig. 2a) and the mean rate of degradation (Fig. 2b and Supplementary Fig. 1) were sensitive to the Mg^2+^ ion concentration. The degradation occurred more slowly at lower concentrations of Mg^2+^, and the normalized fraction of degradation along the time trajectory showed a clear decrease in the degradation rate at lower Mg^2+^ concentrations (Fig. 2c and Supplementary Fig. 2). However, the slower degradation at low Mg^2+^ concentrations was not due to the loss of cleavage activity of exonuclease because almost all DNA substrates were cleaved by the end of the reaction (Supplementary Fig. 3). The saturated enzymatic rate measured in this study (~17 nt s^−1^ at 9 mM MgCl_2_) was consistent with those from bulk biochemical studies^27,30^ (10–12 nt s^−1^) and other single-molecule studies^21–23,31^ (13–18 nt s^−1^). Remarkably, we found a significant reduction in the degradation rate with 15 mM MgCl_2_ (Fig. 2b, red circle).

Interestingly, clear patterns of pauses displaying constant FRET values over an extended period of time were predominantly observed at low Mg^2+^ concentrations (e.g., blue windows in 2^nd^ through 5^th^ panels from top to bottom in Fig. 2a). Detailed analysis reveals that the pause population and its dwell time decreased dramatically with increasing Mg^2+^ concentration (Fig. 2d, e). When the enzyme degrades the FRET-reporting region between two fluorophores, the enzyme exclusively performs processive degradation because the enzyme is topologically engaged by the previously produced 3′ non-hydrolyzed 23-nt-long DNA strand^21^, which is longer than the footprint of the enzyme. This molecular coupling enables ruling out the possibility that the pauses are the consequence of protein dissociation^21^. The velocity versus protein concentration (Supplementary Fig. 4) reveals a protein-concentration independent tendency, suggesting that pauses indeed are not due to protein dissociation. The pauses are most plausibly caused by the dissociation of the Mg^2+^ cofactor from the active site, as conjectured for other two-metal-ion systems^9,32,33^. If Mg^2+^ ions always remain intact at the catalytic site of the enzyme, then the pause dynamics of the enzyme should not change with Mg^2+^ concentration.

### Inhibition of λ-exonuclease activity by Ca^2+^

**Fig. 3:**
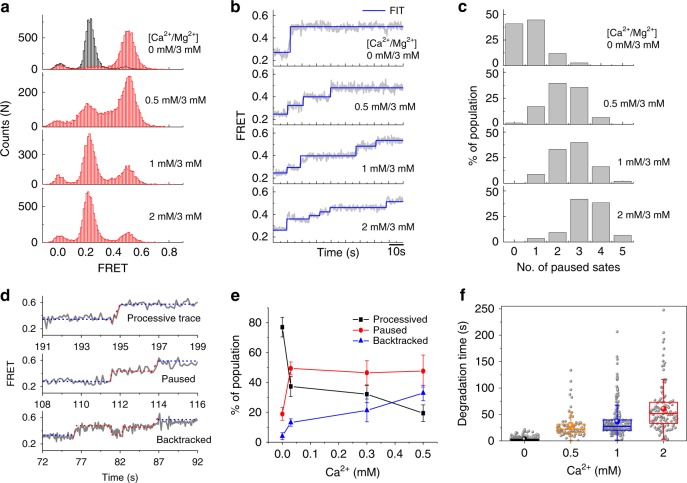
Inhibition of λ-exonuclease activity by Ca^2+^ ions. **a** Single-molecule FRET histograms with various concentrations of Ca^2+^ at the fixed concentration of 3 mM Mg^2+^. **b** Ca^2+^-substitution-induced pause behavior fitted with an automated step-finding algorithm. **c** Pause histograms obtained from various Ca^2+^ concentrations at the fixed concentration of 3 mM Mg^2+^. **d** Three degradation patterns with pauses and backtracking when inhibited by Ca^2+^. **e** The proportion of pause and backtracking as a function of Ca^2+^ concentration. **f** Increase in the degradation time with increasing concentration of Ca^2+^

To verify the foregoing hypothesis that the Mg^2+^ ion dissociates during the reaction, we performed a kinetic poisoning experiment by introducing a catalytically inactive ion, Ca^2+^, to the saturated Mg^2+^ concentration of ~3 mM (Fig. 3a and Supplementary Figs. 5-7). If Mg^2+^ ever dissociates from the exonuclease, Ca^2+^ can replace it, causing the enzymatic reaction to pause because of competitive binding of Ca^2+^ and Mg^2+^ to the active site^6,20^. In the presence of Ca^2+^ ions, a significant number of traces demonstrate pauses (Fig. 3b, c) and backtracking (Fig. 3d, e). The analysis of single-molecule FRET time trajectories in the presence of Ca^2+^ revealed three types of degradation patterns (processive, pause, and backtracking) (Fig. 3d), and the individual FRET time trajectories obtained from different ratios of Ca^2+^ to Mg^2+^ were in quantitative agreement with our conjecture. The increased Ca^2+^/Mg^2+^ ratio reduced the number of processive traces but increased the number of paused and backtracked traces (Fig. 3e), which substantiates the idea that both traces originated from the inhibitory effect of Ca^2+^. The patterns of backtracking are attributed to the backsliding of the enzyme along the undigested single strand due to re-annealing of DNA at the ss/ds junction^20,21^. The degradation time, measured in the presence of both 2 mM Ca^2+^ and 3 mM Mg^2+^, increased ~21 fold relative to that of the case without Ca^2+^ (Fig. 3f). The Ca^2+^ poisoning experiment, which demonstrates the Ca^2+^-concentration-dependent pause behavior, points to the dynamic nature of Mg^2+^ coordination at the active site. Mg^2+^ indeed dissociates upon cleavage.

### Structural clues for the two-metal-ion dynamics

The active sites of the two crystal structures of exonuclease, which were co-crystalized with the DNA substrate and Mg^2+^ or Ca^2+^, offer molecular insight into the metal-ion coordination with the enzyme. The trimeric enzyme has three active sites and retains a PD-(D/E)XK metal-binding motif at the core of its active site consisting of two conserved acidic residues (D/E) and one positive residue (K), conserved in type II endonucleases (Supplementary Fig. 8a, zoomed-in view)^20^. The two metal ions (Mg_A_^2+^ and Mg_B_^2+^) are bridged by D119 and the scissile phosphate of DNA (red circle, left in Supplementary Fig. 8a) in octahedral geometry^20^. One of the two water molecules, chelated to Mg_A_^2+^, nucleophilically attacks the scissile phosphate (Supplementary Fig. 8a, zoomed-in view) upon activation by K131. In contrast, Mg_B_^2+^ stabilizes the trigonal bi-pyramidal geometry, resulting in the formation of the transition state through its coordination with three water molecule, two oxygen atoms of the scissile phosphate, and D119^20^. Among the three subunits of the trimeric ring, only the DNA-bound subunit (green, left in Supplementary Fig. 8a) possesses the two metal ions (blue balls). This result suggests that a DNA substrate is required to both stably bind Mg^2+^ and enable the catalytically competent coordination of two Mg^2+^ ions (Supplementary Fig. 8a, zoomed-in view). As observed in previous studies, bi-metal-ion catalysis becomes viable only in the presence of a substrate with which an enzymatically competent complex is formed^34–36^. In contrast, in the Ca^2+^-bound X-ray structure^6^, Ca^2+^ is stably coordinated even without a DNA substrate (see Ca^2+^ in all the orange, blue, and green subunits: red circles around blue balls, left in Supplementary Fig. 8a). In general, the two-metal-ion catalysis of nucleic acid hydrolysis utilizes the two ions as follows: Mg_A_^2+^ ties to one oxygen atom of the scissile phosphate together with the hydrolytic water molecule that executes the nucleophilic attack, while Mg_B_^2+^ binds to two oxygen atoms of the scissile phosphate^11^.

The new terminal generated upon cleavage (small red dotted circle, zoomed-in view of Supplementary Fig. 8a) is translocated to the original terminal position (large green dotted circle), presumably through electrostatic attractions between the phosphate group and the positively charged pocket of the protein upon product release, as suggested by previous studies^20,25^. With this molecular insight, we hypothesize the following. The translocation of the cleaved phosphate completely breaks the coordination bonds of Mg_B_^2+^ with D119 and the scissile phosphate, which may result in the release of Mg_B_^2+^ during each round of enzymatic turnover. In contrast, Mg_A_^2+^ is likely to remain intact, coordinated by three stationary residues (D119, E129 and L130) at the active site (Supplementary Fig. 8a).

### Kinetic model for the two-metal dynamics

**Fig. 4:**
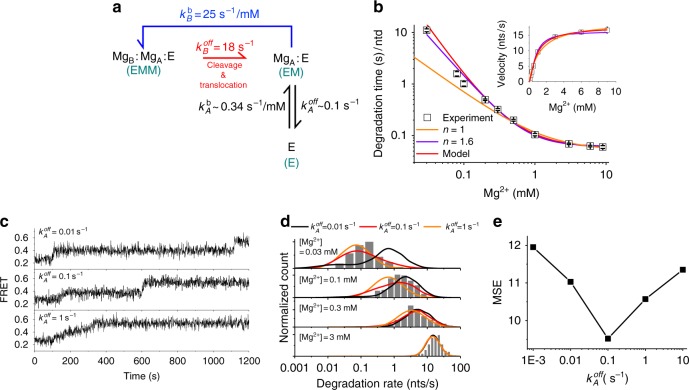
Quantitative analysis of two-metal dynamics. **a** A kinetic model for the two-metal-ion dynamics consisting of three states: *EMM* (exonuclease:Mg_B_^2+^:Mg_A_^2+^ complex), *EM* (exonuclease:Mg_A_^2+^ complex), and *E* (exonuclease only). In the model, Mg_B_^2+^ dissociates upon DNA cleavage and translocation ($k_B^{off}$, red arrow, *EMM* to *EM*) whereas Mg_A_^2+^ dissociates stochastically ($k_A^{{\mathrm{off}}}$, *EM* to *E*). The single cycle is completed upon rebinding of Mg_B_^2+^ ($k_B^b$, *EM* to *EMM*). **b** The degradation time per nucleotide (*τ*_1_) versus Mg^2+^ concentrations. The data are fitted to three models: (1) Michaelis Menten equation (orange line), (2) Hill equation (purple line, *n* = 1.6), and (3) Eq. 1 for the model shown in **a** (red line). Inset shows velocity ( = $\tau _1^{ - 1}$) versus Mg^2+^ concentrations data and their fits (solid lines). **c** Representative FRET time traces from simulations performed at three different choices of $k_A^{off}$: 0.01 *s*^−1^ (top panel), 0.1 *s*^−1^ (middle panel), and 1 *s*^−1^ (bottom panel). [Mg^2+^] is set to 0.03 mM. **d** Degradation rate histograms at four different [Mg^2+^] conditions: 0.03 mM, 0.1 mM, 0.3 mM, and 3 mM. Gray bars represent experimental data, whereas solid lines are theoretical prediction calculated under three difference choices of $k_A^{off}$: 0.01 *s*^−1^ (black), 0.1 *s*^−1^ (red), and 1 *s*^−1^ (orange). **e** Mean squared error (MSE) versus $k_A^{off}$. The errors are calculated by summing the squares of the difference between the experimental and theoretically predicted results

Based on the FRET experimental data and the preceding arguments, we propose a minimal kinetic model that can fully delineate the effect of the metal-ion dynamics on the activity of λ-exonuclease (Fig. 4a). The model consists of three states: (1) *EMM* (exonuclease:Mg_A_^2+^:Mg_B_^2+^ complex), (2) *EM* (exonuclease:Mg_A_^2+^ complex), and (3) *E* (exonuclease). Mg_B_^2+^ dissociates (*EMM* to *EM*) at a rate of $k_B^{{\mathrm{off}}}$, whereas Mg_B_^2+^ rebinds (*EM* to *EMM*) as described by a single rate constant $k_B^{{\mathrm{on}}} = k_B^{\mathrm{b}}\left[ {{\mathrm{Mg}}^{2 + }} \right]$. The dissociation (*EM* to *E*) and rebinding (*E* to *EM*) of Mg_A_^2+^ are described by $k_A^{{\mathrm{off}}}$ and$k_A^{{\mathrm{on}}} = k_A^{\mathrm{b}}\left[ {{\mathrm{Mg}}^{2 + }} \right]$, respectively. Because the translocation-coupled duplex melting is the rate-limiting step^20,23^, the cleavage and translocation were modeled as a single step transition.

In the proposed model, the degradation rate per nucleotide (Fig. 4b. See also Fig. 2b) varies with [Mg^2+^] (Eq. 1, see the methods for the derivation):

1 $$V\left( {\left[ {{\mathrm{Mg}}^{2 + }} \right]} \right) = \frac{{k_B^{{\mathrm{off}}}\left[ {{\mathrm{Mg}}^{2 + }} \right]}}{{\left[ {{\mathrm{Mg}}^{2 + }} \right] + K_B + K_BK_A\left[ {{\mathrm{Mg}}^{2 + }} \right]^{ - 1}}} = \tau _1^{ - 1}$$

where $K_B = k_B^{{\mathrm{off}}}/k_B^b,K_A = k_A^{{\mathrm{off}}}/k_A^b,$ and τ_1_ is the mean degradation time per nucleotide. The experimental data are well fitted to Eq. 1 over the entire range of [Mg^2+^] (Fig. 4b, red line), yielding $k_B^{{\mathrm{off}}} = 18\,{\rm{s}}^{- 1},K_B = 0.71\,{\rm{mM}}$, and *K*_*A*_ = 0.30 mM, thus $k_B^b = 25\,{\rm{s}}^{- 1}\left( {{\rm{mM}}} \right)^{ - 1}$(Fig. 4b). At high [Mg^2+^] (»*K*_*A*_), Eq. 1 approximates to the standard Michaelis-Menten (MM) equation. Indeed, the experimental data were well fitted for high [Mg^2+^], (e.g., [Mg^2+^] > *K*_*B*_( = 0.71 mM) but deviated significantly from MM kinetics at low [Mg^2+^], ruling out the possibility of a super-stable Mg_A_^2+^ coordination. Without the two-ion dissociation, the deviation of *V* at low [Mg^2+^] (<0.1 mM) from the MM kinetics cannot be explained. Alternatively, the Hill equation,$V\left( {\left[ {{\mathrm{Mg}}^{2 + }} \right]} \right) = k_B^{{\mathrm{off}}}\left[ {{\mathrm{Mg}}^{2 + }} \right]^n/\left( {\left[ {{\mathrm{Mg}}^{2 + }} \right]^n + K_B^n} \right)$ with n = 1.6 describes the data well (violet line in Fig. 4b). The Hill coefficient of n = 1.6, higher than 1, suggests that two ions bind semi-cooperatively; that is, Mg_A_^2+^ must be present in the active site for Mg_B_^2+^ to bind.

The minor difference between *K*_*B*_ and *K*_*A*_ (*K*_*B*_/*K*_*A*_ = 2.4), corresponding to the stability difference of <1 k_B_T, is somewhat unexpected given that the coordination number of Mg_A_^2+^ to the surrounding residues is greater than that of Mg_B_^2+^ (i.e., 3 to 1 as in Supplementary Fig. 8a). However, very different kinetic constants can give the same thermodynamic stability. To estimate $k_A^{{\mathrm{off}}}$, which best explains the experimental time traces, we compare histograms of the degradation velocity (Fig. 4d) collected from experiments with those generated from the simulation at different $k_A^{{\mathrm{off}}}$. Representative FRET time traces from simulations are shown in Fig. 4c. We found that the simulation results obtained with $k_A^{{\mathrm{off}}}$ = 0.1 *s*^−1^ best reproduce the trend in the experimental data (Fig. 4e and Supplementary Fig. 9). We also compared mean pause times and their histograms (Supplementary Fig. 10a, b). Again, the simulation results obtained with $k_A^{{\mathrm{off}}}$ = 0.1 *s*^−1^ most closely match the experimental data trend (Supplementary Fig. 10c). Furthermore, simulated time traces at varying [Mg^2+^] with $k_A^{{\mathrm{off}}}$ = 0.1 *s*^−1^ (Supplementary Fig. 11) show a gradual decrease in the pausing dwell time and an increase in the translocation velocity as a function of [Mg^2+^], which also closely resemble the experimental observations (Fig. 2b and Fig. 4b). Based on these results, we conclude $k_A^{{\mathrm{off}}} \approx 0.1\,{\rm{s}}^{- 1}$ and $k_A^{\mathrm{b}} \approx 0.34\,{\rm{s}}^{- 1}\left( {{\rm{mM}}} \right)^{ - 1}$.

### Dynamic coupling between metal-ion stability and degradation

**Fig. 5:**
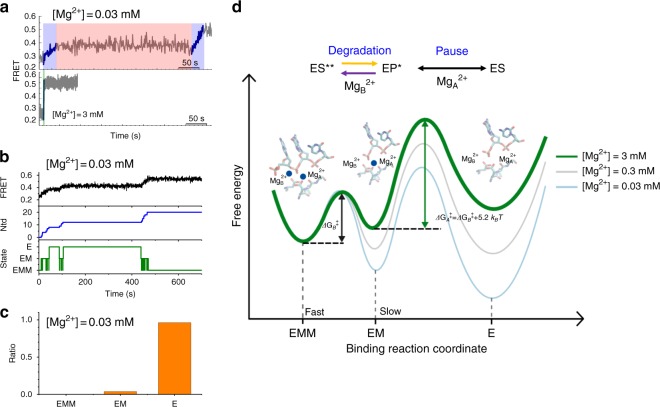
FRET time trajectories and the free energy landscape during metal-ion dynamics. **a** Two experimental FRET time trajectories, showing Mg_B_^2+^-dynamics-dependent slope change (purple) and a pause caused by the dissociation of two metal ions, Mg_A_^2+^ and Mg_B_^2+^ (pink). **b** A representative simulation trajectory, displaying three panels: a time-FRET trace (black line in the top panel), degradation position of exonuclease along DNA (the line in the middle panel), and its metal-ion state (green line in the bottom panel). The trajectory revealed that iteration between *EM* and *EMM* decreases the degradation slope in a Mg^2+^-dependent manner, and pauses occur due to the *E* state by dissociation of both metal ions. **c** Total fraction of single-molecule traces showing the pause state at three different Mg^2+^ states. **d** Free energy landscape along the reaction coordination of Mg^2+^ binding and dissociation. Processive degradation occurs during a repetitive cycle between the 1^st^ and 2^nd^ metal-ion binding states, whereas a pause occurs due to the dissociation of two metal ions. The energy barrier between the 2^nd^ and 1^st^ states is lower than the one between the 1^st^ and 2^nd^ states due to the different numbers of ligands surviving after the translocation (e.g., 1 versus 3 ligands with protein residues for Mg_B_^2+^ and Mg_A_^2+^, respectively). Transitions between ES** and EP* are indicated by two one-directional arrows to emphasize the transitions are non-reversible and not in equilibrium

A representative trajectory from the simulations at [Mg^2+^] = 0.03 mM shows a clear signature of pauses (black in the top panel in Fig. 5b) along with the number of cleaved nucleotides (blue in the middle panel) and the chemical states of exonuclease over time (green in the bottom panel). Comparing the three panels in Fig. 5b clarifies that the transitions from *EMM* to *EM* or between *EM* and *E* slow down the degradation process (Fig. 5b). A careful inspection of the FRET time trajectories at varying [Mg^2+^] suggests that the distinct pause (pink window in Fig. 5a and top window in Fig. 5b) arises from the dissociation of both Mg_A_^2+^ and Mg_B_^2+^, especially at low [Mg^2+^]. More than 95% of the observed paused states are caused by the trapping in the *E* state (Fig. 5c). In contrast, a slope change from steep to mild that corresponds to a decrease in translocation speed results from the binding and dissociation of Mg_B_^2+^ alone (compare the top and bottom windows in Fig. 5a, shaded in purple and green, respectively). More time trajectories based on the simulations and the analysis of different [Mg^2+^] are available in Supplementary Fig. 12. This simulation indicates that if Mg_B_^2+^ alone dissociates and rebinds during each round of catalysis, then long and frequent pauses would not arise; instead, degradation would be smooth. We examined a model based on simultaneous dissociation of both Mg_A_^2+^ and Mg_B_^2+^ as well, but found that it could not generate the marked pauses (Supplementary Fig. 13).

Whereas Mg_A_^2+^ and Mg_B_^2+^ bind to the active site with similar ion-coordination stability (i.e., they have similar binding constant, *K*_*A*_≈*K*_*B*_), the Mg_A_^2+^ ion both binds and unbinds from the active site significantly more slowly because the Mg_A_^2+^ ion is coordinated by more residues than the Mg_B_^2+^ ion at the active site (Supplementary Fig. 8a). The difference between the kinetic barriers to the dissociation of Mg_B_^2+^ (${\mathrm{\Delta G}}_B^\ddagger$) and Mg_A_^2+^ (${\mathrm{\Delta G}}_A^\ddagger$) is estimated as ${\mathrm{\Delta G}}_A^\ddagger - {\mathrm{\Delta G}}_B^\ddagger \approx k_BT\log \left( {k_B^{{\mathrm{off}}}/k_A^{{\mathrm{off}}}} \right) \approx 5.2\,k_BT$, indicating that the Mg_A_^2+^ ion dissociates ~200 times more slowly than Mg_B_^2+^. Indeed, a comparison of the MD-simulated electrostatic interactions of Mg_A_^2+^ and Mg_B_^2+^ with their molecular environment reveals that Mg_A_^2+^ ions are more strongly held by the surrounding residues (Supplementary Fig. 14 and SI text), lending support to the molecular insight inferred from the crystal structure (Supplementary Fig. 8a).

**Fig. 6:**
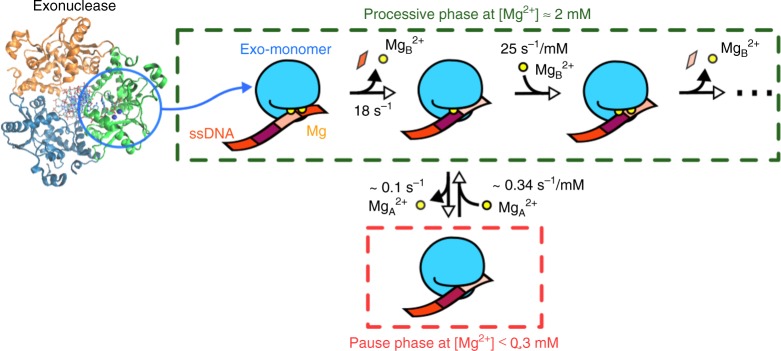
The mechanism of two-metal-ion dynamics. The active site of one subunit of the homotrimer is shown in green whereas two Mg^2+^ ions and nucleotides are represented by yellow circles and rectangles in various colors, respectively. Metal-ion coordination to the catalytic active site is highly dynamic so that two Mg^2+^ ions (Mg_A_^2+^ and Mg_B_^2+^) can coordinate with and dissociate from the surrounding ligands. Mg_A_^2+^ remains stably bound to the active site, but Mg_B_^2+^, which is close to the 5′ terminal side of the scissile phosphate, dissociates during every round of catalytic cleavage. As a result, the dissociation of Mg_B_^2+^ facilitates product release and exonuclease translocation, promoting the overall processivity of exonuclease activity. More specifically, at high [Mg^2+^] (≈2 mM), λ-exonuclease degrades processively and its cleavage activity is mainly controlled by Mg_B_^2+^ dynamics (green dashed square). The fast unbinding/rebinding dynamics of Mg_B_^2+^ and strict requirement of two Mg^2+^ ions for the catalytic step yield a Mg^2+^-concentration-dependent exonuclease activity. Conversely at low [Mg^2+^] (≤0.3 mM), occasional unbinding of Mg_A_^2+^ from the catalytic site and slow rebinding of Mg_A_^2+^ stalls the exonuclease activity, giving rise to a long pause (red dashed square), thus elucidating the molecular origin of dynamic heterogeneity in exonuclease activity. The dynamic variation in the coordination states of two metal ions orchestrates the multistep process of exonuclease activity

A mechanistic insight into metal-ion dynamics in exonuclease activity was acquired based on single-molecule FRET time trajectories, Mg^2+^-bound high-resolution crystal structures, and a careful comparison of FRET data with the simulation results derived from the kinetic model. Mg_B_^2+^ dissociates during each round of catalysis due to the translocation, but Mg_A_^2+^ dissociates only occasionally. As implied by the inter-conversions of *EM* ↔ *EMM* in the bottom panel of Fig. 5b (the green line), Mg_B_^2+^ dissociates whenever a transition is made from *EMM* to *EM*. Consequently, the rate of DNA degradation alters in a [Mg^2+^]-dependent manner. The occasional dissociation of Mg_A_^2+^ (transition from *EM* to *E*), albeit ~200-fold slower than the dissociation of Mg_B_^2+^ (transitions from *EMM* to *EM*), gives rise to a long pause. As summarized in Fig. 6, metal-ion dynamics orchestrate enzymatic activity in the following manner: at low Mg^2+^ concentrations, rebinding of Mg^2+^ becomes rate-limiting, slowing down the overall degradation rate; at physiological Mg^2+^ concentrations, the dissociation of Mg_B_^2+^ during each round of cleavage facilitates product release and the translocation, optimizing the enzymatic turnover rate for multiple rounds of hydrolysis; and Mg^2+^ concentrations above 9 mM suppress the probability of vacancy and Mg_B_^2+^ dissociation (red circle in Fig. 2b), thus preventing product release and translocation for the next round of cleavage.

## Discussion

The coordination of metal ions to the active site of λ-exonuclease displays distortion from the perfect octahedral geometry (Supplementary Fig. 8a), suggesting that they are in an energetically unfavorable state. If both metal ions ever dissociate, then it will take a longer time to re-assemble the functionally competent DNA–protein complex because the allosteric coordination of the two ions inevitably incurs a higher kinetic penalty than that of a single ion. This explains why the two metal ions bind in a sequential manner, displaying cooperativity during the enzymatic reaction. Although the ion-coordination stabilities of Mg_A_^2+^ and Mg_B_^2+^ are comparable, there is substantial difference in their kinetic barriers associated with both binding and dissociation, as illustrated in Fig. 5d.

The difference of ~5.2 *k*_*B*_*T* between the kinetic barriers for dissociation of Mg_A_^2+^ and Mg_B_^2+^ (Fig. 5d), estimated from our theoretical model, indicates that Mg_A_^2+^ dissociates ~200 times more slowly than Mg_B_^2+^. Thus, frequent Mg_B_^2+^ dissociation for fast product release while keeping Mg_A_^2+^ bound to prevent a large conformational change to an inactive state allows exonucleases to achieve high processivity at physiological [Mg^2+^] (~3–9 mM). Furthermore, the long pauses due to the dissociation of two metal ions may act as an additional regulatory mechanism at a low [Mg^2+^] (<0.3 mM). In other words, the dissociation of Mg_B_^2+^ is not random but occurs in a cleavage and translocation-dependent manner. Given that the translocation of the enzyme for the next cleavage is limited by the rate of product release (<50 ms), an excessively stable coordination of Mg_B_^2+^ would hinder product release, slowing down the degradation process, as evidenced by the slower translocation velocity at a high [Mg^2+^] (~15 mM). (Fig. 2b, red circle).

Biochemical and structural analyses ^25,29,32–34^ of RNase H found that after cleavage, the scissile phosphate could no longer simultaneously coordinate the two Mg^2+^ ions, suggestive of cooperative binding right after the cleavage. The high-resolution crystal structures^16,32,37^ and QM/MM MD simulations^11^ of RNase H also suggest a cooperative effect of the two metal ions but this cooperation has never been kinetically demonstrated by time-resolved experiments on a relevant time scale. Our data clearly demonstrate the cooperative effect of two metal ions, as shown in the sigmoidal curves (Figs. 2b, 4b), which point to the allosteric conformational changes during the enzymatic activation. Furthermore, the long pause indirectly demonstrates their cooperativity and that both metal ions are definitely required to form a catalytically competent DNA–protein complex. Even though our kinetic fitting into the Hill function yields *n* = 1.6, suggesting that at most two metal ions participate in the function of exonuclease, there might be another transient intermediate in which a third metal-ion shortly binds and leaves a site of enzymes, other than the two canonical metal-binding sites, as indicated by the recent structural^7–9^ and computational^38–40^ studies. It is widely believed that metal ion cofactors are stably bound to the catalytic sites of enzyme, and that their lifetimes are much longer than enzymatic cycles owing to the concept that the coordination with chelating ligands is thermodynamically stable. However, our study clarifies that the timing of metal-ion coordination and dissociation from the active site is one of the key factors determining the rates of the enzymatic cycle. Our finding provides experimental kinetic evidences on fluctuations of the metal-ion coordination during enzyme activity, and offers a detailed insight to the type II nuclease, opening to future dynamic studies on other two-metal-ion catalysis system. The single-molecule assay developed here enables deciphering the role of metal-ion dynamics in enzymatic cycles through the time trajectories of cleavage-coupled translocation along DNA. Based on all the data, we propose a full enzymatic cycle: (1) catalytically active DNA-protein complex formation (after initiation), (2) cleavage, (3) product and Mg_B_^2+^ release upon translocation with concomitant melting, (4) metal-ion rebinding, (5) then a return to step 1. Our study elucidates the full effect of Mg^2+^ dynamics on processive activity of λ-exonuclease (Figs. 2 and 5); clarifies a molecular basis for metal-ion dynamics at the active site of the enzyme and provides mechanistic insight into the origin of the dynamic heterogeneity in enzymatic activity, which has been reported previously for λ-exonuclease^23^ as well as other proteins and nucleic acids^41–50^.

## Methods

### Protein expression and purification

The λ-exonuclease gene was amplified by PCR from genomic DNA of bacteriophage λ (D3654-5UN, Sigma Aldrich) by using primers designed for ligation-independent cloning^25,51^. The PCR product was treated with T4 DNA polymerase and cloned into the pB4 ((his)_6_-tag-Maltose Binding Protein- TEV site) vector. The cloned vector was confirmed by DNA sequencing. The vector was transformed into BL21-Star (DE3) *E. coli* (Thermo Fisher Scientific) and λ-exonuclease was expressed in 1 L of LB medium. Bacterial cultures were grown to an OD_600_ of 0.5, at which time IPTG was added at a final concentration of 0.3 mM. After shaking for 3.0 h at 37 °C, bacteria were harvested by centrifugation at 5000 × *g*, re-suspended in 20 mL of buffer (50 mM Na phosphate pH 8.0, 5 mM Tris, 300 mM NaCl, supplemented with EDTA-free protease inhibitor cocktail) and lysed by sonication. The cell lysate was clarified by centrifugation for 30 min at 35,000 × *g*. His-MBP-tagged λ-exonuclease was purified by nickel affinity chromatography (His-Trap FF, GE Healthcare). His-MBP-tag was removed by TEV protease, and native λ-exonuclease was collected in the flow-through by the second nickel affinity chromatography. The purified native protein was dialyzed and stored in a buffer (25 mM Tris-HCl (pH8.0), 50 mM NaCl, 1 mM DTT, 0.1 mM EDTA, and 50% glycerol).

### DNA substrate preparation

DNA oligonucleotide strands used for single-molecule FRET experiments were purchased from Integrated DNA Technologies (IDT). The DNA substrate used to probe the processive phase was constructed by ligating two pieces of DNA at room temperature for 1 h using T4 ligase (#M0202, New England Biolabs) and purified on a 15% PAGE gel. The sequences and modifications can be found in the supplementary information.

### Single-molecule assays

DNA constructs were tied on a quartz surface coated with PEG (Laysan Bio) to minimize nonspecific surface adsorption of proteins^29,52^. We immobilized ~50 pM DNA molecules to the imaging chamber to achieve an appropriate density for single-molecule imaging. The reaction buffer contained 67 mM glycine-KOH (pH 9.4), various concentrations of MgCl^2^, 50 μg ml^−1^ BSA, 1 mg ml^−1^ Trolox (Sigma-Aldrich) and an oxygen-scavenging system of 1 mg ml^−1^ glucose oxidase (Sigma- Aldrich), 0.04% mg ml^−1^ catalase (Sigma-Aldrich) and 0.4% (w/v) D-glucose (Sigma- Aldrich). Trolox was employed as a triplex-state quencher to avoid fluorescent photo-blinking, and glucose and glucose oxidase were used to remove oxygen, which triggers rapid photo-bleaching of fluorescent dyes^29,52^. The reaction began by injection of the reaction buffer containing λ-exonuclease into DNA molecules at room temperature.

### Single-molecule data acquisition

FRET donor (Cy3) on the DNA was excited by a green laser (532 nm, 100 mW, Coherent Compass Laser). The fluorescence emission light from Cy3 and Cy5 was collected by a water immersion objective lens (UPlanApo 60×, Olympus) and then cleaned by a 550 nm long-pass fluorescence filter equipped in a total internal reflection fluorescence (TIRF) microscopy. The emission light was divided into donor and acceptor signals with a 635 nm dichroic mirror (Chroma) and was recorded by iXon Ultra 897 EMCCD camera (Andor). Both recorded fluorescence intensities of Cy3 and Cy5 were in an arbitrary unit (a.u.) since they were amplified by a gain factor. The data were saved in a video file format by a software written in Visual C++. Fluorescence intensities of single molecules were extracted by IDL software and FRET efficiency was calculated as the ratio of intensities, Accepter Intensity/(Donor Intensity + Acceptor Intensity) after amending cross-talk between the donor and acceptor channels. All data were analyzed with MATLAB codes and plotted in Origin software.

### Protein-Mg^2+^ degradation simulation: mean velocity

The mean degradation time for a single nucleotide *τ*_1_ of the model shown in Fig. 4a can be expressed as

2 $$\tau _1 = \frac{1}{{k_B^{{\mathrm{off}}}}} + \tau _d + \frac{1}{{k_B^{{\mathrm{on}}}}}$$

The first term on the right is the mean transition time for *EMM*→*EM*, where *τ*_*d*_ is the average dwell time in either the *EM* or *E* state, and the final term denotes the mean transition time of *EM*→*EMM*. Competition between the two transitions *EM*→*E* and *EM*→*EMM* determines *τ*_*d*_, which can be written as

3 $$\tau _d = \frac{{k_A^{{\mathrm{off}}}}}{{k_A^{{\mathrm{off}}} + k_B^{{\mathrm{on}}}}}\frac{1}{{k_A^{{\mathrm{on}}}}} + \left( {\frac{{k_A^{{\mathrm{off}}}}}{{k_A^{{\mathrm{off}}} + k_B^{{\mathrm{on}}}}}} \right)^2\frac{1}{{k_A^{{\mathrm{on}}}}} + \left( {\frac{{k_A^{{\mathrm{off}}}}}{{k_A^{{\mathrm{off}}} + k_B^{{\mathrm{on}}}}}} \right)^3\frac{1}{{k_A^{{\mathrm{on}}}}} + \ldots = \frac{{k_A^{{\mathrm{off}}}}}{{k_A^{{\mathrm{on}}}k_B^{{\mathrm{on}}}}}$$

The first term represents the escape of exonuclease to *EMM* from *EM* after only one excursion to the E state, whereas the second term describes two excursions to the *E* state before escaping to *EMM*, etc. Hence, we obtain

4 $$\tau _1 = \frac{1}{{k_B^{{\mathrm{off}}}}}\left( {1 + \frac{{k_A^{{\mathrm{off}}}k_B^{{\mathrm{off}}}}}{{k_A^{{\mathrm{on}}}k_B^{{\mathrm{on}}}}} + \frac{{k_B^{{\mathrm{off}}}}}{{k_B^{{\mathrm{on}}}}}} \right) = \frac{1}{{k_B^{{\mathrm{off}}}\left[ {{\mathrm{Mg}}^{2 + }} \right]}}\left( {\left[ {{\mathrm{Mg}}^{2 + }} \right] + \frac{{K_BK_A}}{{\left[ {{\mathrm{Mg}}^{2 + }} \right]}} + K_B} \right)$$

Finally, we obtain the expression of mean velocity *V*=1/*τ*_1_, which is used in the main text to fit the [Mg^2+^]-dependent data for *V* (Fig. 4b).

### Velocity histogram analysis and $k_A^{{\mathrm{off}}}$ estimation

The velocity histograms show a large heterogeneity (Supplementary Fig. 9), which may originate from the presence of dynamic disorder, i.e., the previously observed heterogeneity in rate constants^23^. Although the rate constant of the single exonuclease itself can change within a single time trajectory, we assume that such an event is rare as the DNA sample that was degraded in this study was only 20 nt long. We rather consider the scenario of quenched disorder, i.e., the difference between the rate constants among individual time traces.

To incorporate the heterogeneity in the analysis, we assume that each rate constant can be expressed as

5 $$\begin{array}{l}k_B^{{\mathrm{off}}} = k_B^{{\mathrm{off}},{\mathrm{o}}}e^{ - {\mathrm{\delta \Delta }}G^\ddagger },k_B^{\mathrm{b}} = k_B^{{\mathrm{b}},{\mathrm{o}}}e^{ - {\mathrm{\delta \Delta }}G^\ddagger },k_A^{\mathrm{b}} = \\ k_A^{{\mathrm{b}},{\mathrm{o}}}e^{ - {\mathrm{\delta \Delta }}G^\ddagger },k_A^{{\mathrm{off}}} = k_A^{{\mathrm{off}},{\mathrm{o}}}e^{ - \delta {\mathrm{\Delta }}G^\ddagger }\end{array}$$

where *δ*Δ*G*^‡^ represents the variation in the activation free energy from one molecule to another. Here, $k_B^{{\mathrm{\xi }},{\mathrm{o}}}$’s denotes the disorder-free rate constants. $k_B^{{\mathrm{off}},{\mathrm{o}}}$ and $k_B^{b,o}$ are determined from the [Mg^2+^] versus *V* data (Fig. 4b). $k_A^{{\mathrm{b}},{\mathrm{o}}}$ is determined once we find $k_A^{{\mathrm{off}},{\mathrm{o}}}$ by using *K*_*A*_ estimated from [Mg^2+^] versus *V* data. Since *δ*Δ*G*^‡^, contributed by the disorder, results from the sum of many interactions between protein residues, we assume that *δ*Δ*G*^‡^ follows the normal distribution with zero mean and standard deviation *σ*^2^. Enzymes without disorder lead to *σ* = 0. To estimate *σ*^2^ of exonuclease, we fit the log *V* histograms to a Gaussian distribution (Supplementary Fig. 9, green lines).

Next, to estimate $k_A^{{\mathrm{off}},{\mathrm{o}}}$, we first calculate the probability density function (PDF) of degradation time for 20 nts, *τ*20, with randomly generated *δ*Δ*G*^‡^ for a given $k_A^{{\mathrm{off}},{\mathrm{o}}}$. The PDF is calculated numerically by solving the following set of differential equations describing the kinetic model proposed in Fig. 4a.

6 $$\begin{array}{l}\frac{{dP_{EMM}\left( {n,t} \right)}}{{dt}} = \hskip 0pt - k_B^{{\mathrm{off}}}P_{EMM}\left( {n,t} \right)\left( {1 - \delta _{20,n}} \right) \\ \hskip 75pt+ \, k_B^{{\mathrm{on}}}P_{EM}\left( {n - 1,t} \right)\left( {1 - \delta _{0,n - 1}} \right)\end{array}$$

7 $$\begin{array}{l}\frac{{dP_{EM}\left( {n,t} \right)}}{{dt}} = k_B^{{\mathrm{off}}}P_{EMM}\left( {n,t} \right) - \left( {k_B^{{\mathrm{on}}} + k_A^{{\mathrm{off}}}} \right)P_{EM}\left( {n,t} \right)\\ \hskip -65pt+ k_A^{{\mathrm{on}}}P_E\left( {n,t} \right)\end{array}$$

8 $$\frac{{dP_E\left( {n,t} \right)}}{{dt}} = k_A^{{\mathrm{off}}}P_{EM}\left( {n,t} \right) - k_A^{{\mathrm{on}}}P_E\left( {n,t} \right)$$

where $k_B^{{\mathrm{on}}} = k_B^{\mathrm{b}}\left[ {{\mathrm{Mg}}^{2 + }} \right]$, $k_A^{{\mathrm{on}}} = k_A^{\mathrm{b}}\left[ {{\mathrm{Mg}}^{2 + }} \right]$. *P*_*S*_(*n*,*t*) denotes the probability that the exonuclease is in the state *S*, where *S* ∈ {*E*,*EM*,*EMM*}  is positioned at the *n*-th nucleotide at time *t*, where *n*∈{0,1,2,…,20}. For the numerical integration, the *expm* function in *MATLAB* was employed. To calculate the probability density functions that retain molecule-to-molecule heterogeneity, we generated 5000 random numbers for *δ*Δ*G*^‡^ and took the average over them. Next, we calculated the PDF of ${\mathrm{\zeta }} \equiv \log _{10}V = \log _{10}\left( {\frac{{20}}{{\tau _{20}}}} \right)$ by using the following transformation.

9 $$P\left( \zeta \right) = P\left( {\tau _{20}} \right)\left| {\frac{{d\tau _{20}}}{{d\zeta }}} \right| = \tau _{20} \times \log 10 \times P\left( {\tau _{20}} \right)$$

The mean square error between the experimentally obtained velocity histograms and the theoretical prediction is minimized when $k_A^{{\mathrm{off}}} = 0.1\,s^{ - 1}$ (Fig. 4e). The relatively large error in the fit at high [Mg^2+^] (black, red, orange lines in Supplementary Fig. 9) arises from the difference in the arithmetic mean of V ($= 1/{\sum} {\tau _i}$, where τ_i_ is the degradation time obtained from *i*-th time trace), which is used in the estimate of rate constants ($k_B^{{\mathrm{off}}},k_B^{\mathrm{b}},K_A$) in Fig. 4b, and the arithmetic means of log *V* of histograms.

### Generation of simulation data

The synthetic time traces were generated using the Gillespie algorithm^53^ (Fig. 4c).

### Pause time analysis of simulation data

The pause times from the simulated time traces were collected when the exonuclease was stalled in the same nucleotide (i.e., when the enzyme showed no degradation activity) for longer than 2 s (Supplementary Fig. 10b). To compare the experimental and simulated pause times, we used only pause times longer than 10 sec (Supplementary Fig. 10b, black dashed line). The 10 s threshold was selected to minimize the error in the experimental pause times, because the noise inherent to the FRET signal hinders the unambiguous detection of short pause events.

### Coincident dissociation of two Mg^2+^ do not yield long pauses

As exonuclease and associated DNA undergo significant structural changes upon cleavage and translocation step, it is possible that both Mg_A_^2+^, Mg_B_^2+^ ions dissociate at every cleavage event. To further clarify the stability of Mg_A_^2+^ ion upon cleavage event, we develop another model (model-2, Supplementary Fig. 13a).

Compared to the previous model (model-1, Fig. 4a), a cleavage of DNA and subsequent translocation of exonuclease in the model-2 are always accompanied with the dissociation of both Mg_A_^2+^ and Mg_B_^2+^ (*EMM* to *E*, Fig. Supplementary Fig. 13a, red arrow). Subsequent reversible binding and dissociation of Mg_A_^2+^ (*E* to *EM*, *EM* to *E*) and rebinding of Mg_B_^2+^ (*EM* to *EMM*) in the model-2 are identical with the model-1. Again, here we posit based on crystal structure that the stable binding of Mg_B_^2+^ requires the binding of Mg_A_^2+^ before.

In the model-2, at least one Mg_A_^2+^ rebinding event is necessary for each enzymatic cycle. Thus, it is expected that, unlike the model-1, if rebinding of Mg_A_^2+^ is slow, long pause is expected even at high concentration of Mg^2+^ ( > 0.3 mM) which is not observed experimentally. This implies that in model-2, $k_A^{\mathrm{b}}$ » 0.1 and long-pause is not expected even at low Mg^2+^ concentration.

To quantitatively check the conclusion above, we perform kinetic analysis and simulation. The degradation rate per nucleotide again has the same mathematical form with Eq. 1 in main text but the interpretation of fitting parameters changes.

10 $$V\left( {\left[ {{\mathrm{Mg}}^{2 + }} \right]} \right) = \frac{{k_B^{{\mathrm{off}}}}}{{1 + \left( {K_A + K_B} \right)/\left[ {{\mathrm{Mg}}^{2 + }} \right] + K_BK_A/\left[ {{\mathrm{Mg}}^{2 + }} \right]^2}}$$

where $K_B = k_B^{{\mathrm{off}}}/k_B^b,K_A = k_A^{{\mathrm{off}}}/k_A^b$ as before. The equation can be derived by the same approach used for model-1. Again, $k_B^{{\mathrm{off}}}$, *K*_*A*_, and *K*_*B*_ are estimated from the fit (Fig. 4b) and $k_A^b,k_B^b$ can be written as function $k_A^{{\mathrm{off}}}$ but in more complicated form.

11 $$k_A^b = \frac{{\left( {K_A + K_B} \right)k_A^{{\mathrm{off}}} - \sqrt {k_A^{{\mathrm{off}}^{\mathrm{2}}}\left( {K_A + K_B} \right)^2 - 4k_B^{{\mathrm{off}}}k_A^{{\mathrm{off}}}\left( {K_AK_B} \right)} }}{{2K_AK_B}}$$

12 $$k_B^b = \frac{{\left( {K_A + K_B} \right)k_A^{{\mathrm{off}}} + \sqrt {k_A^{{\mathrm{off}}^{\mathrm{2}}}\left( {K_A + K_B} \right)^2 - 4k_B^{{\mathrm{off}}}k_A^{{\mathrm{off}}}\left( {K_AK_B} \right)} }}{{2K_AK_B}}$$

Here, we assume ${\mathrm{k}}_{\mathrm{A}}^b \le {\mathrm{k}}_{\mathrm{B}}^b$ based on the crystal structure. The above equations implies ${\mathrm{k}}_{\mathrm{A}}^{{\mathrm{off}}} \ge \frac{{4K_AK_B\hskip .1ptk_B^{{\mathrm{off}}}}}{{\left( {K_A + K_B} \right)^2}} = 31.4\,\mathrm{s}^{ - 1}$ which mathematically supports our qualitative conclusion $k_A^{\mathrm{b}}$ » 0.1 in the previous paragraph.

We further test our conclusion with kinetic simulation with ${\mathrm{k}}_{\mathrm{A}}^{{\mathrm{off}}} = 32,64,$ and 128 s^−1^. Generated FRET time traces using the model-2 (Supplementary Fig. 13b), pause-time histograms (Supplementary Fig. 13c), and their mean square error to experimental data (Supplementary Fig. 13d) imply that long paused state of exonuclease is not observed in the model-2.

### Futile ion binding

Frequent bindings and dissociations of Mg^2+^ ions but with many futile events are plausible. In this case, our model (model-1, Fig. 4a) can be deemed as the simplest model that describes futile binding and the occasional progress to a stable binding state by means of kinetic rate constant ($k_A^b$ and $k_B^b$).

## Electronic supplementary material

Supplementary Information

## Data Availability

Data supporting the findings of this manuscript are available from the corresponding authors upon reasonable request.
