# Supplementary material for: Dynamic coordination of two-metal-ions orchestrates λ-exonuclease catalysis
Source: Nat Commun. 2018 Oct 23;9:4404. doi: 10.1038/s41467-018-06750-9 (PMC6199318; doi:10.1038/s41467-018-06750-9)
Supplement: Supplementary file 1 — Supplementary Information [file 41467_2018_6750_MOESM1_ESM.docx]

**SUPPLEMENTARY INFORMATION:**

**Dynamic Coordination of Two-Metal-Ions, One Transient and the Other Stable, Orchestrates Multistep Activity of λ***-***Exonuclease**

Wonseok Hwang^2,4,#^, Jungmin Yoo^1,#^, Yuno Lee^2,5^, Suyeon Park^1^, Phuong Lien Hoang^1^, HyeokJin Cho^1^, Jeongmin Yu^1^, Thi Minh Hoa Vo^1^, Minsang Shin^3^, Mi Sun Jin^1^, Daeho Park^1^, Changbong Hyeon^2,^* and Gwangrog Lee^1,^*

^1^School of Life Sciences, Gwangju Institute of Science and Technology, Gwangju, 61005, Republic of Korea

^2^Korea Institute for Advanced Study, Seoul, 02455, Republic of Korea

^3^Department of Microbiology, Kyungpook National University School of Medicine, 680 Gukchaebosang-Ro, Jung-gu, Daegu, 41944, Republic of Korea

^4^Present address: Clova AI Research, NAVER Corp., Seongnam, 13561, Republic of Korea

^5^Present address: Korea Research Institute of Chemical Technology, Daejeon, 34114, Republic of Korea

Supplementary information:

Supplementary Materials and Methods

Supplementary Figures 1-14 and Legends

1. **Materials and Methods**

**Protein and DNA**

λ-exonuclease was expressed and purified using primers (Lambda LIC Forward Primer-JM**:** G GGC GGC GGT GGT GGC GGC ATG ACA CCG GAC ATT ATC C; Lambda LIC Reversed Primer-JM**:**GTT CTT CTC CTT TGC GCC CTC ATC GCC ATT GCT CCC C) designed for ligation-independent cloning[^1^](#_ENREF_1) as described in the methods in the main text. All DNA oligonucleotides were purchased from Integrated DNA Technologies (IDT) and detail sequences and positions of modifications for biotin and fluorescent dyes are provided below.

**For the single-molecule assay:**

3’ strand:

5’-Biotin TGG CGA CGG CAG CGA GGC TTA ATT /iCy5/TGT TAA ATA TGG CGA TTC TC/iCy3/A CGC CAA CAT GTA ATT TAG GCA G -3’: (5’-Biotin TGG CGA CGG CAG CGA GGCTTA ATT /iCy5/TGT TAA ATAT -3’ plus 5’-Phosphate GGC GAT TCT C/iCy3/AC GCC AAC ATG TAATTT AGG CAG -3’)

5’ degradation strand:

5’-phosphate CTG CCT AAA TTA CAT GTT GGC GTG AGA ATC GCC ATA TTT AAC AAA TTA AGC CTC GCT GCC GTC GCC A -3’

**For the degradation gel assay:**

5’-TGG CGA CGG CAG CGA GGC TTA ATT TGT TAA ATA TGG CGA TTC TCA CGC CAA CAT GTA ATT TAG GCA G -3’

5’-phosphate CTG CCT AAA TTA CAT GTT GGC GTG AGA ATC GCC ATA TTT AAC AAA TTA AGC CTC GCT GCC GTC GCC A-/Cy3/-3’

Fluorescently labelled dye positions are denoted by /iCy5/ and /iCy3/. The labelling method was based on the single-molecule techniques laboratory manual[^2^](#_ENREF_2). The labelled oligonucleotide strand and its complementary strand were annealed by heating a 1.5 mL tube that contained a micro-molar concentration of the oligonucleotides to 90°C for 5 minutes in 10 mM Tris-HCl and 50 mM NaCl, pH 8.0, and then cooling for ~3 hours to room temperature.

**Degradation gel assay**

The 5’ degradation strand of the construct was labelled via a 3’ amino modification with Cy3 mono NHS ester (Invitrogen): 5 nmol of the oligo in 35 μl of 50 mM sodium tetraborate buffer pH 8.5 was incubated with 50 nmol of Cy3 by shaking overnight at room temperature. The labelled oligonucleotides were first purified by ethanol precipitation to remove unreacted dyes and then further purified using denaturing PAGE. For the degradation reactions, ~5 pmol of DNA was mixed with 8 pmol λ-exonuclease in 50 μl buffer solution containing 67 mM glycine-KOH, 100 µg/ml BSA (pH 9.4), and various MgCl_2_ or CaCl_2_ concentrations, as indicated. The samples were incubated for different time courses at room temperature, and the reactions were stopped by adding 50 μl of formamide. The reaction products were resolved on a 15% denaturing PAGE gel and imaged using a fluorescence imager (Typhoon, GE Biosciences).

**2. Protein-Mg^2+^ molecular dynamic (MD) simulation**

**All-atom MD simulation**

To compare the binding stabilities of Mg_A_^2+^ and Mg_B_^2+^ with the active site of exonuclease, MD simulations of exonuclease WT and the K131A mutant were performed employing the crystal structure (PDB ID: 3SM4). In the case of the K131A mutant (identical with the original crystal structure 3SM4), the interaction patterns did not change noticeably during the 100 ns simulation time (Fig. S14a). The average non-bonded energy of Mg_A_^2+^ is ~90 kcal/mol lower than that of Mg_B_^2+^ (Fig. S14a red and blue solid lines). In the case of WT, a stronger binding of Mg_A_^2+^ was observed after 40 ns (Fig. S14b) due to the additional interaction with oxygen in the 3rd phosphate group and the K131 residue, which forms a stable salt-bridge interaction with the 5’ oxygen atom of the third nucleotide backbone. The average non-bonded energy of Mg_A_^2+^ in the 100-200 ns region is ~350 kcal/mol lower than that of Mg_B_^2+^ (Fig. S14b red and blue solid lines). Overall, in both K131A and WT cases, Mg_A_^2+^ is more tightly bound to the binding pocket of exonuclease than Mg_B_^2+^, supporting our conclusion that Mg_A_^2+^ is more likely than Mg_B_^2+^ to remain stable upon cleavage.

**MD simulation methods**

The MD simulation of the exonucleases was performed using GROMACS software (ver. 5.1.2)[^3^](#_ENREF_3)^,^ [^4^](#_ENREF_4) with the CHARMM36 force field[^5^](#_ENREF_5) and TIP3P water model. The system box size was set to ~11×11×11 nm^3^ consisting of three exonuclease monomers, dsDNA, two Mg^2+^ ions, and ~39,000 water molecules at 150 mM NaCl concentration. The 5’ terminal phosphate was patched based on the topology of the CHARMM36 all-hydrogen nucleic acid[^6^](#_ENREF_6). The steepest descent energy minimization with a tolerance value of 2,000 kJ mol^-1^nm^-1^ was used to remove bad contacts in the initial structure. The system was equilibrated using position-restrained runs in the NVT ensemble at 300 K for 100 ps and subsequently under the NPT ensemble at 300 K and 1 bar for 100 ps. These production runs of K131A and WT were executed for simulation times of 100 ns and 200 ns, respectively, in the NPT ensemble using the V-rescale thermostat[^7^](#_ENREF_7) and the Parrinello-Rahman barostat^[8](#_ENREF_8)^. The initial structure of WT was constructed based on the final snapshot of the K131A simulation. The short-range van der Waals and electrostatic interactions were calculated with a distance cut-off at 12 Å, and the long-range electrostatics were computed by the particle mesh Ewald (PME) method[^9^](#_ENREF_9). Non-bonded energy was calculated by summing the *van der Waals* and electrostatic energies as mentioned in the Supplementary Figure 13, and the interaction energy between each Mg^2+^ ion and the surrounding atoms from the ion was computed with 12 Å cutoff.[^10^](#_ENREF_10) We used Allner's LJ parameter of Mg^2+^ ion[^11^](#_ENREF_11) which is currently available in CHARMM36 force field.

**
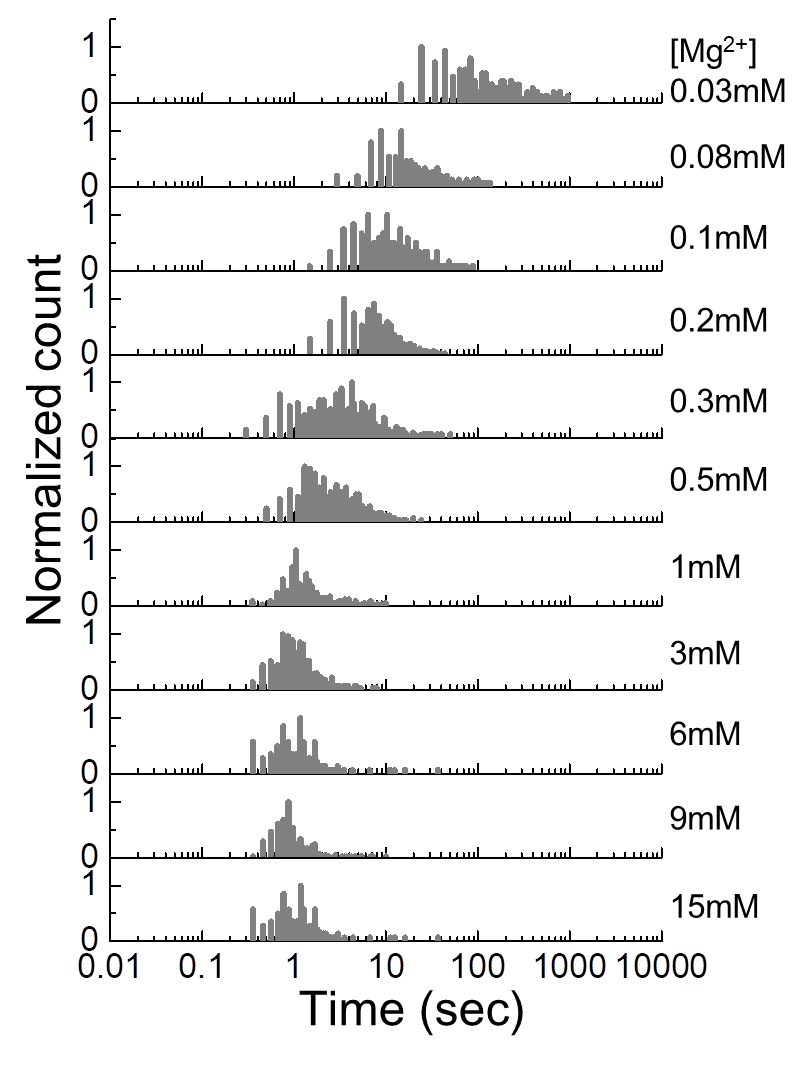
**

**Supplementary Figure 1 |** Distributions of processive degradation time, defined as the time during which FRET increases from the minimum to the maximum levels, as a function of [Mg^2+^].

**
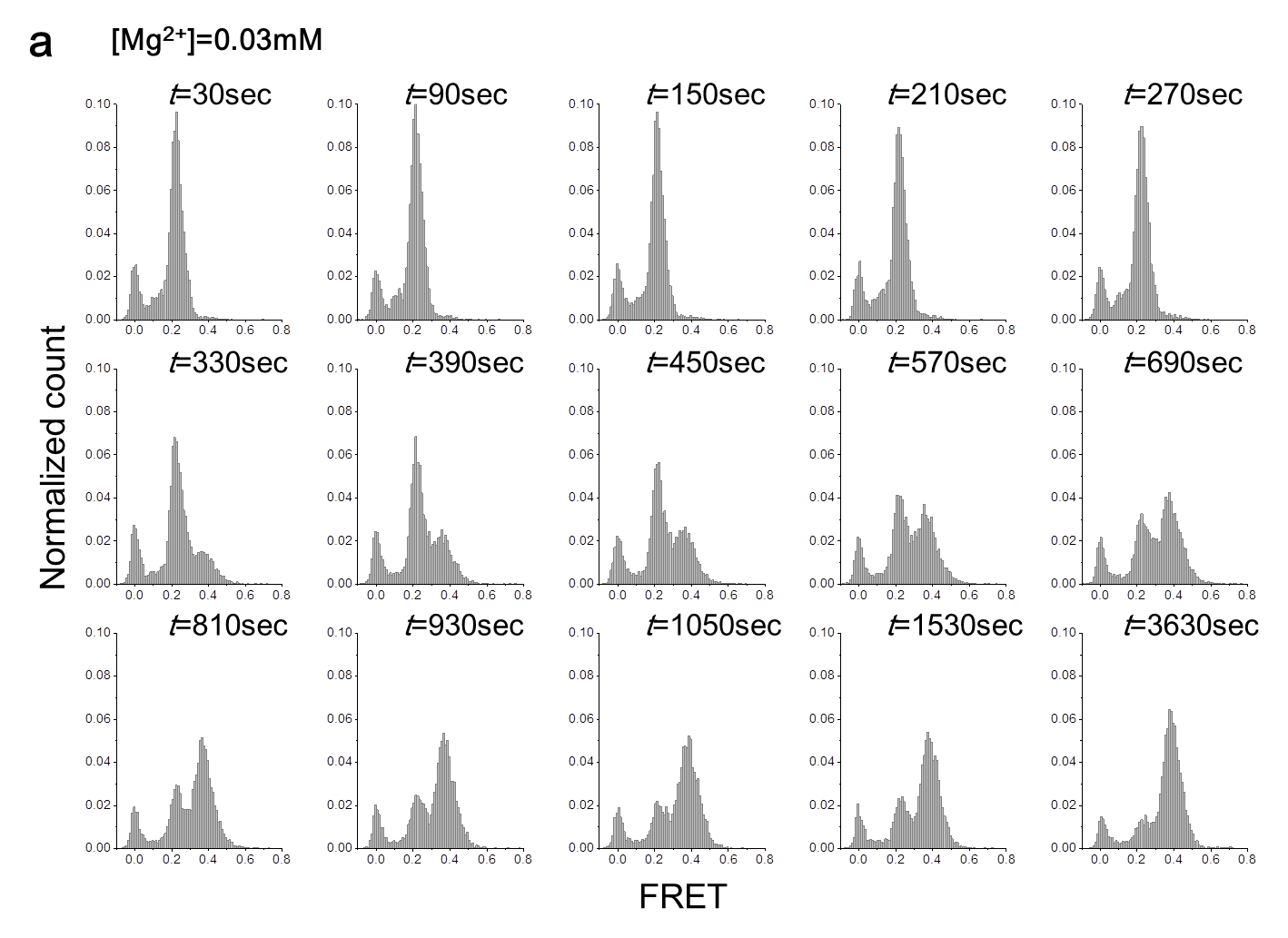
**

**
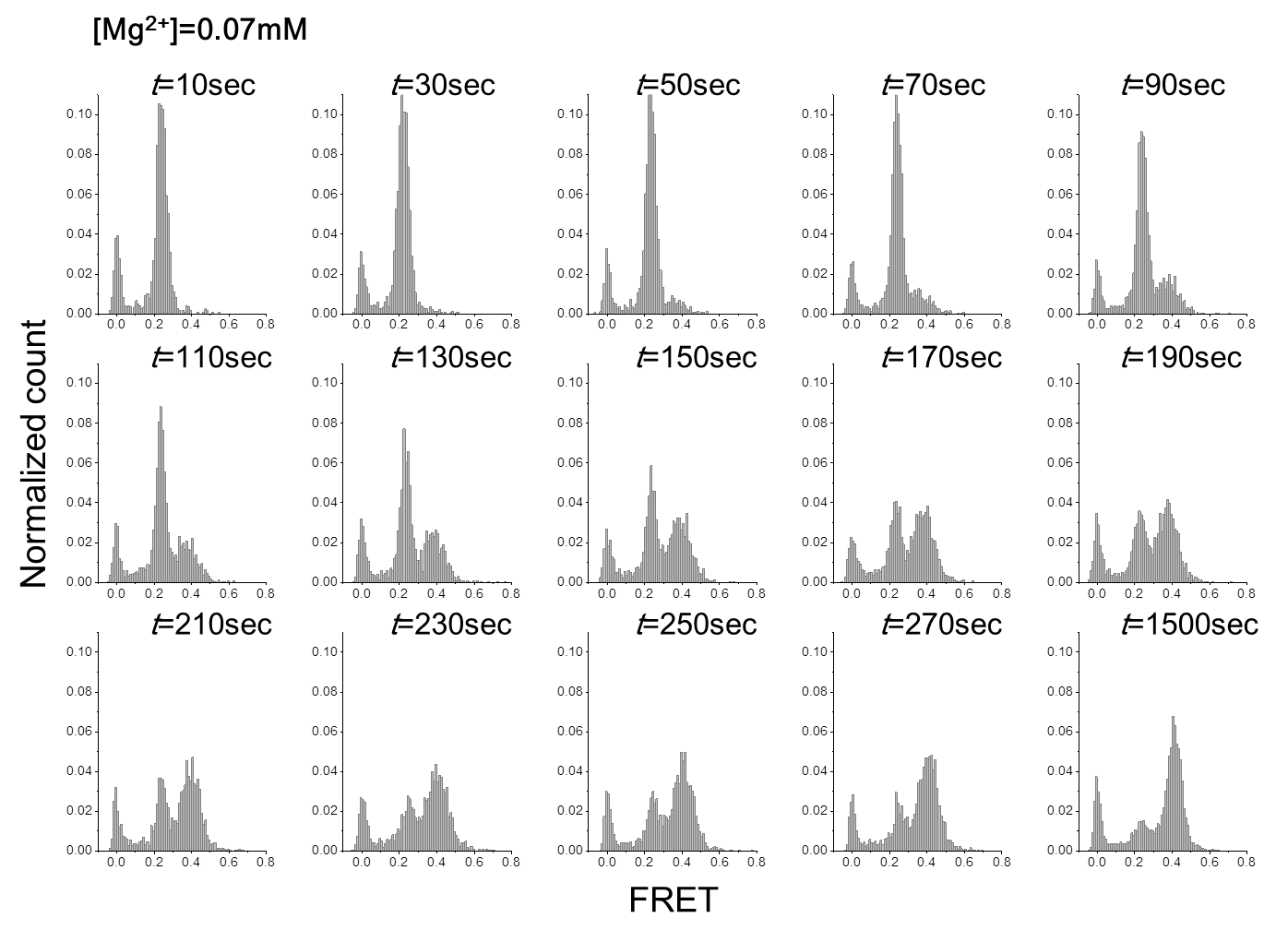
**

**
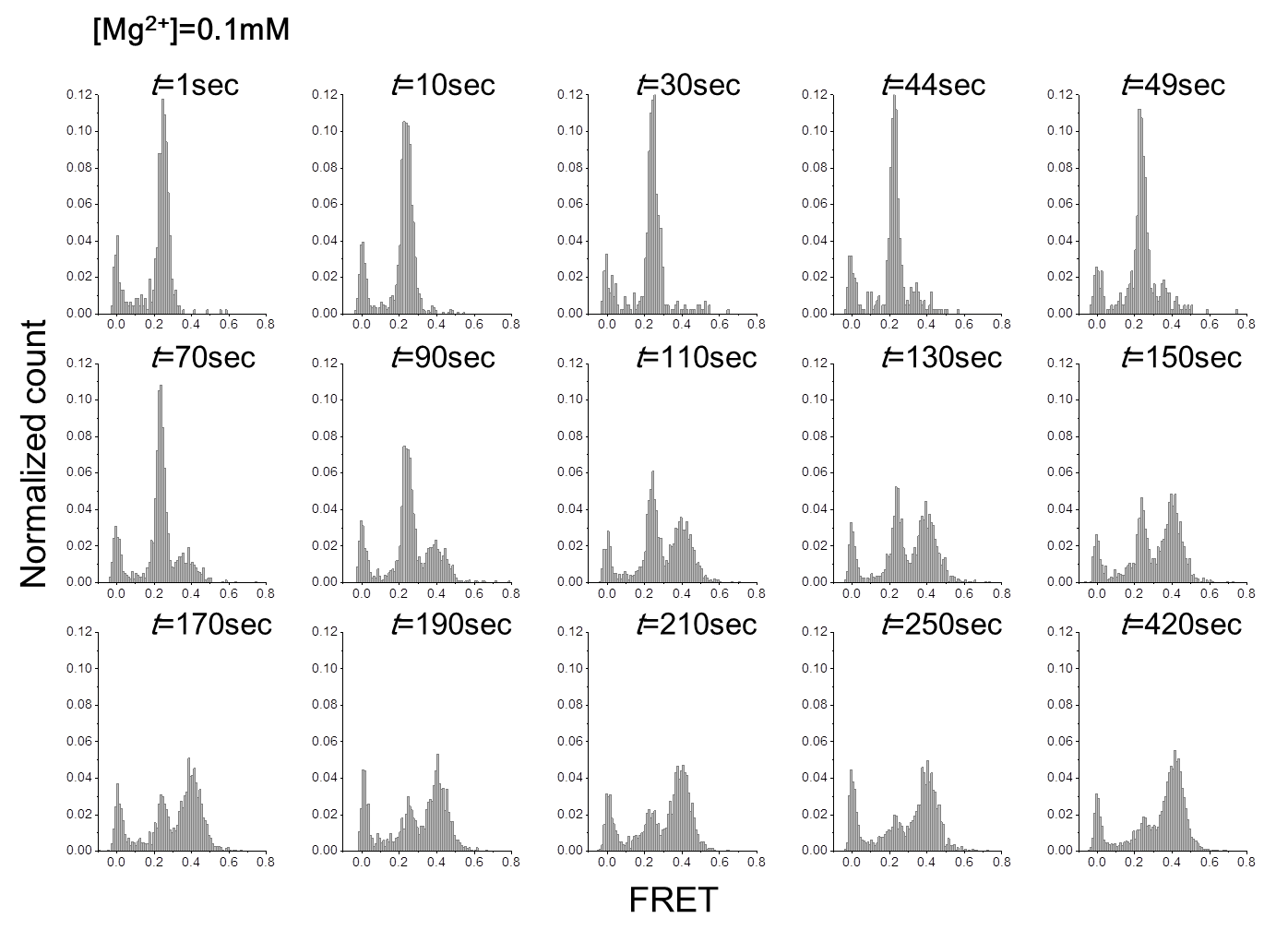
**

**
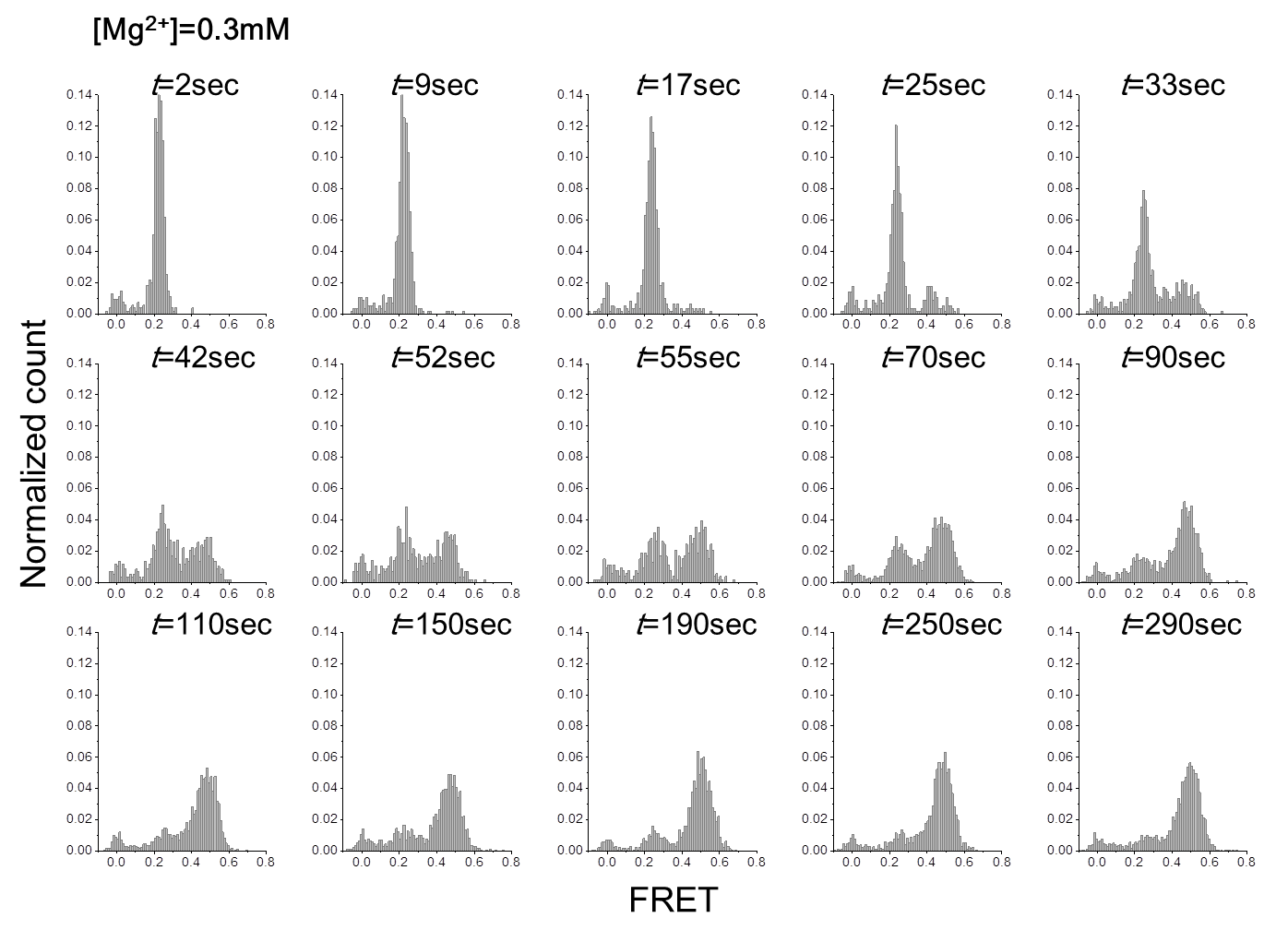
**

**
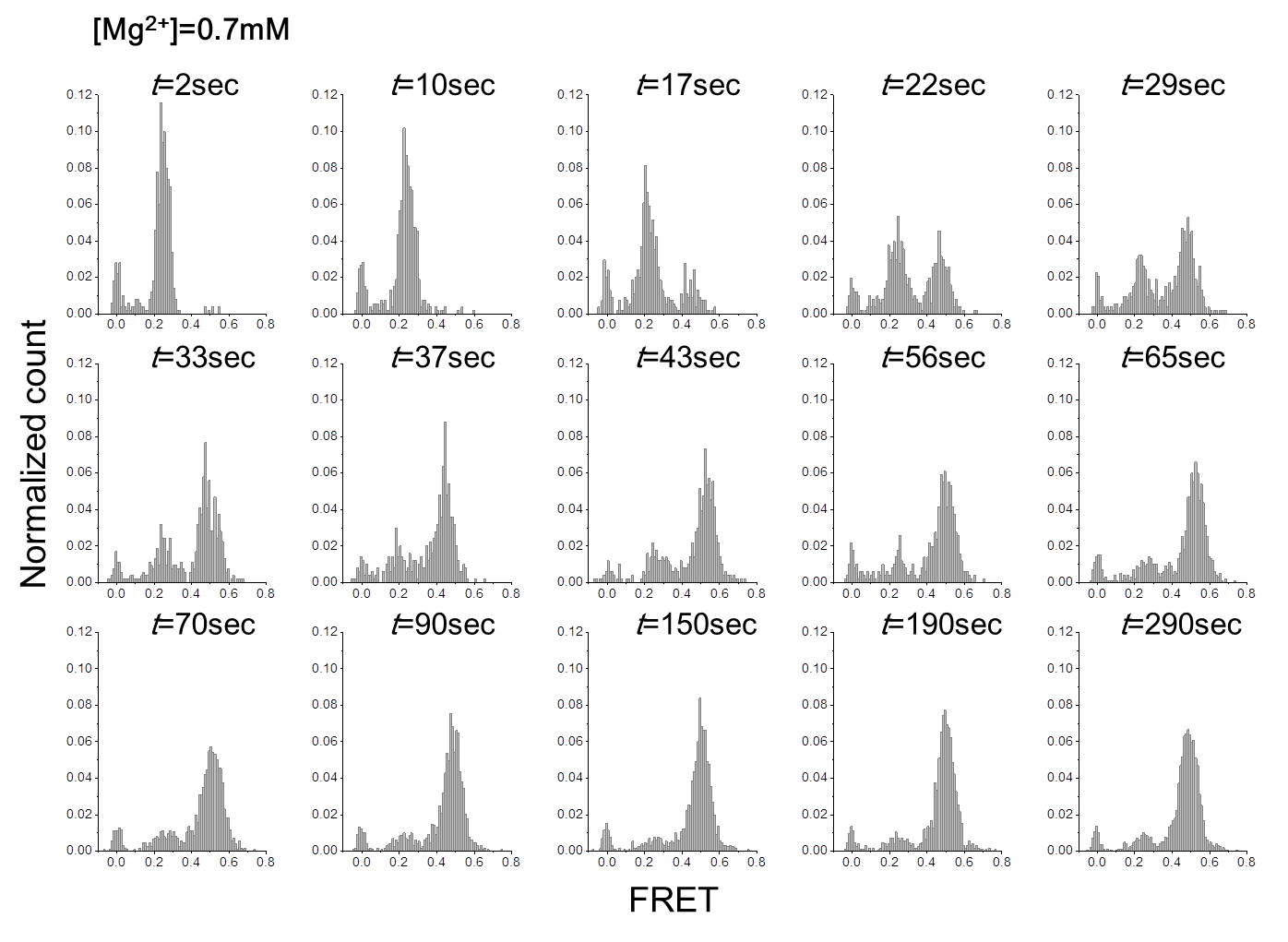
**

**
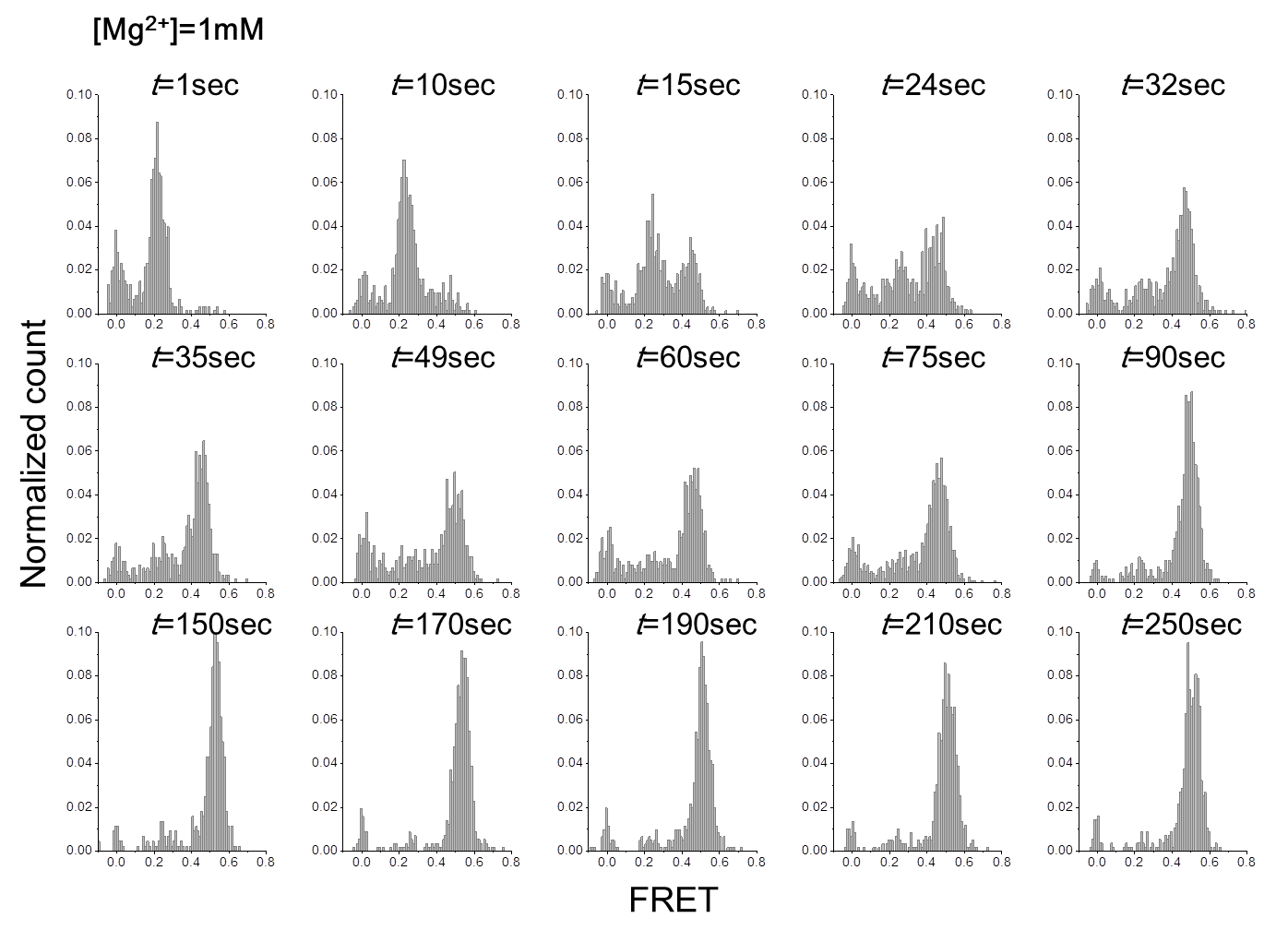
**

**
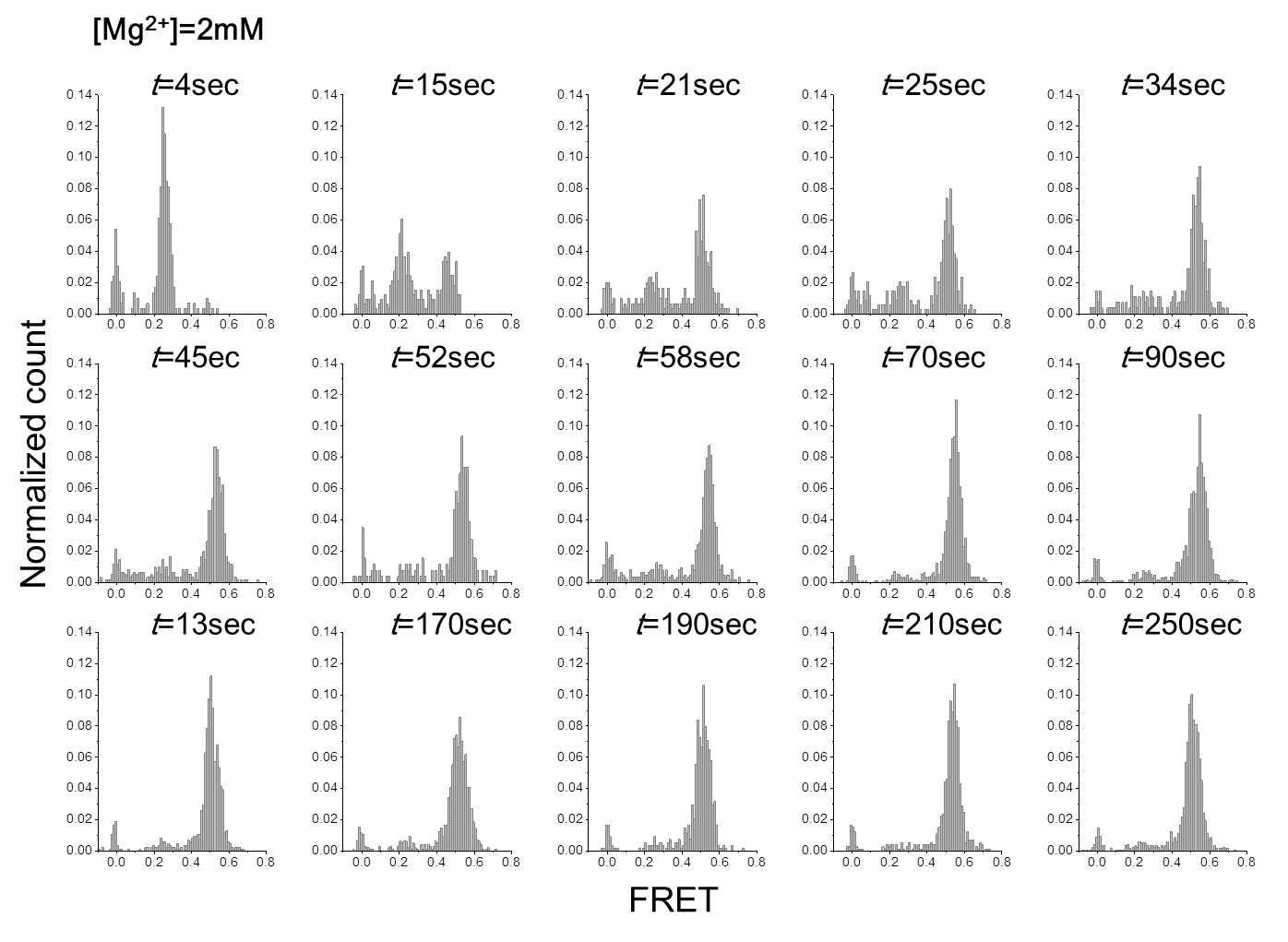
**

**
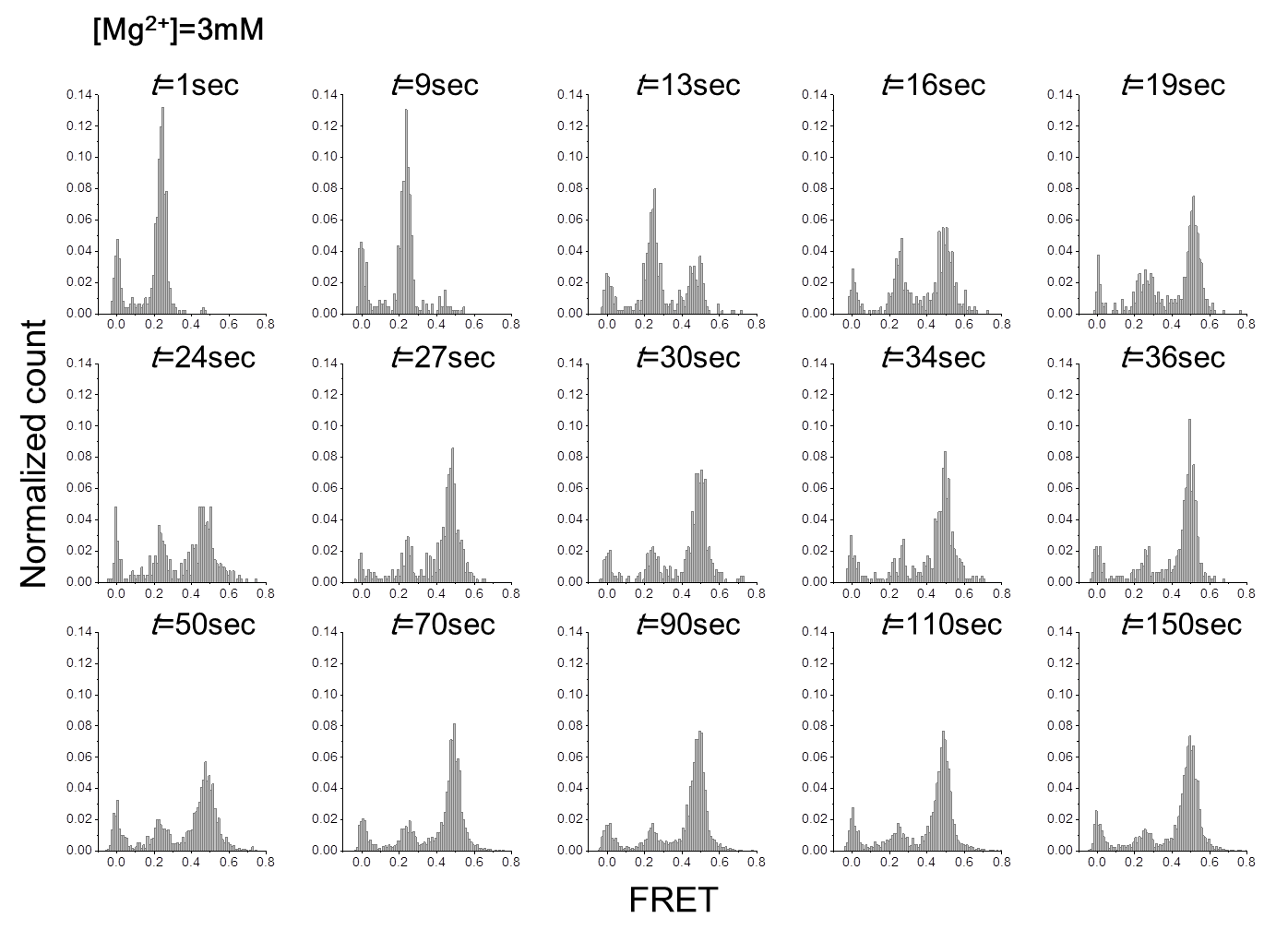
**

**
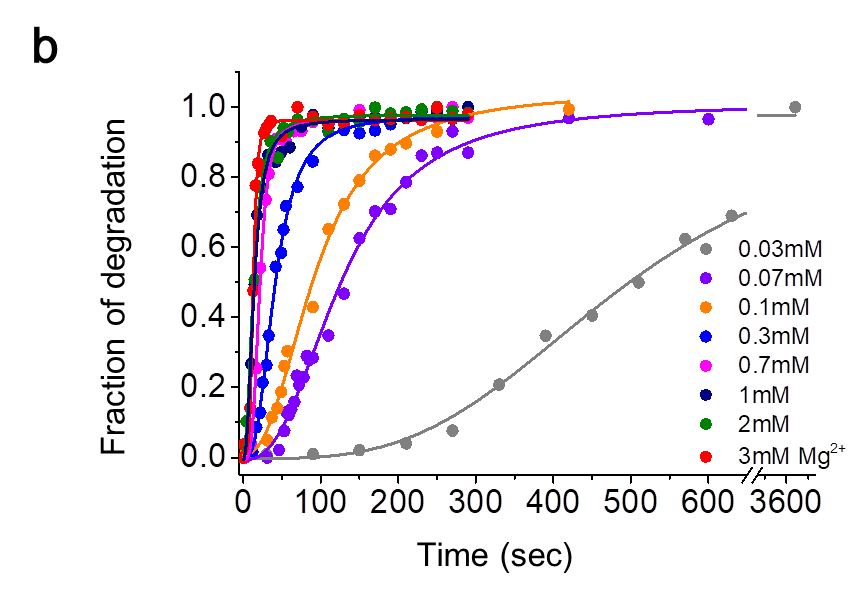
**

**Supplementary Figure 2 |** Mg^2+^ dependence of degradation. **(a)** Time-course histograms obtained from various concentrations of Mg^2+^. **(b)** Fraction of degradation as a function of time at various concentration of Mg^2+^. To plot the rate of fraction of degradation versus Mg^2+^ concentration, the growths of the degradation fraction over the time course were fitted to the logistic equation^[4]^, typically used to calculate EC50 (half maximal effective concentration) at each Mg^2+^ concentration.

**
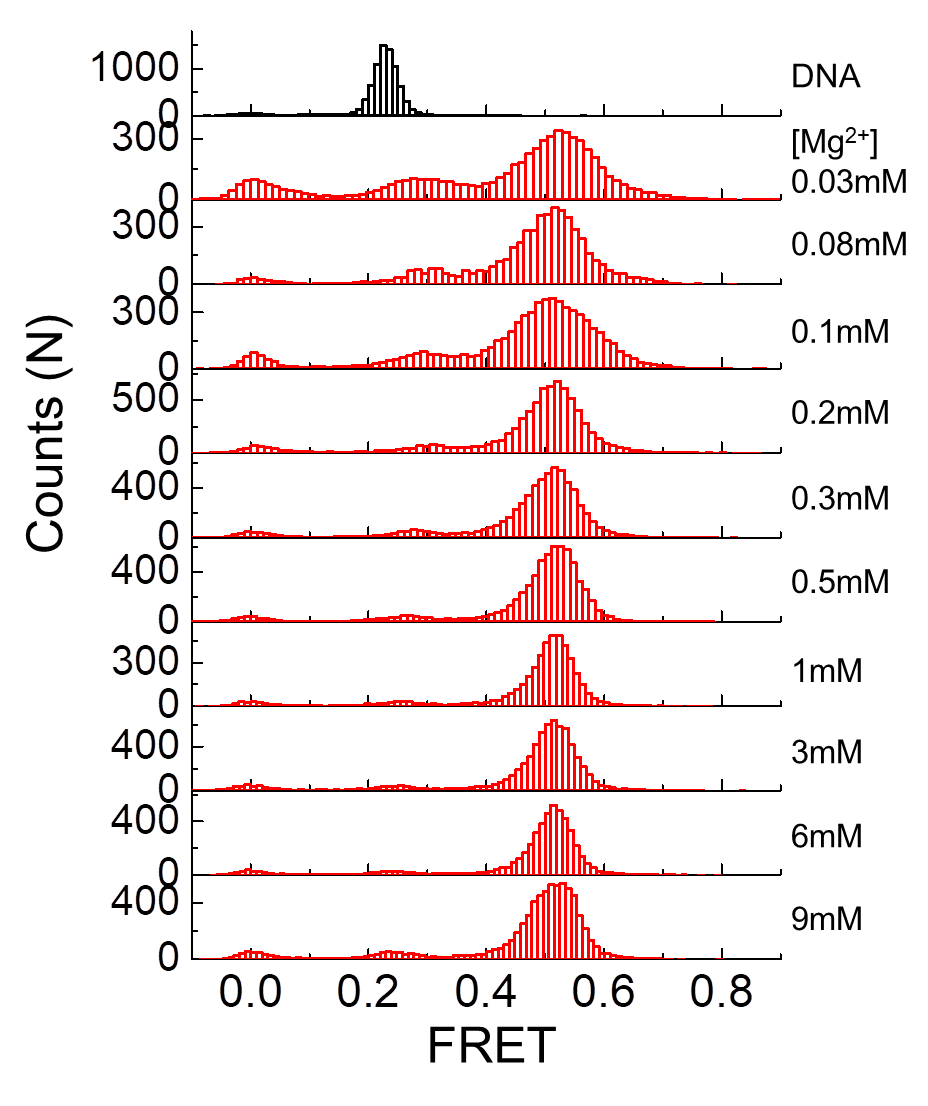
**

**Supplementary Figure 3 |** Single-molecule FRET histograms obtained from various Mg^2+^ concentrations: 1 h (0.03 mM), 10 min (0.08 mM), and 2 min (0.1 to 9 mM) after the reaction.

**Supplementary Figure 4 |** (a) The percentage of pause population versus Mg^2+^ concentration (adopted from main Fig. 2d). We chose 0.1mM Mg^2+^ (red circle) because of the fact that its degradation timescale is appropriate to be free from the fluorescence photo-bleaching issue, providing a reasonable yield, and ~74% of all the trajectories show pauses. (b) Box plots show the distribution of degradation times at various concentrations of λ-exonuclease under 0.1mM Mg^2+^. Boxes represent the interquartile range (IQR); gray balls represent the raw data (based on 172 molecules at 8nM, 707 molecules at 16nM, 245 molecules at 80nM, and 406 molecules at 160nM λ-exonuclease); colored-balls represent the mean values of data; lines within the boxes represent median values of data; and the range of whiskers are 1.5 times than the IQR. (c) Average degradation time as a function of λ-exonuclease concentration. (d) The degradation velocity (i.e., average velocity: 20 nucleotides divided by the average degradation time of c) versus λ-exonuclease concentration under 0.1mM Mg^2+^ reveals a protein-independent tendency (red dotted line of Fig. c) suggesting that indeed the pauses are not due to protein dissociation. The tendency will follow the blue line, if pauses come from dissociation of the enzyme. Error bars denote the standard error of the mean (SEM).

**
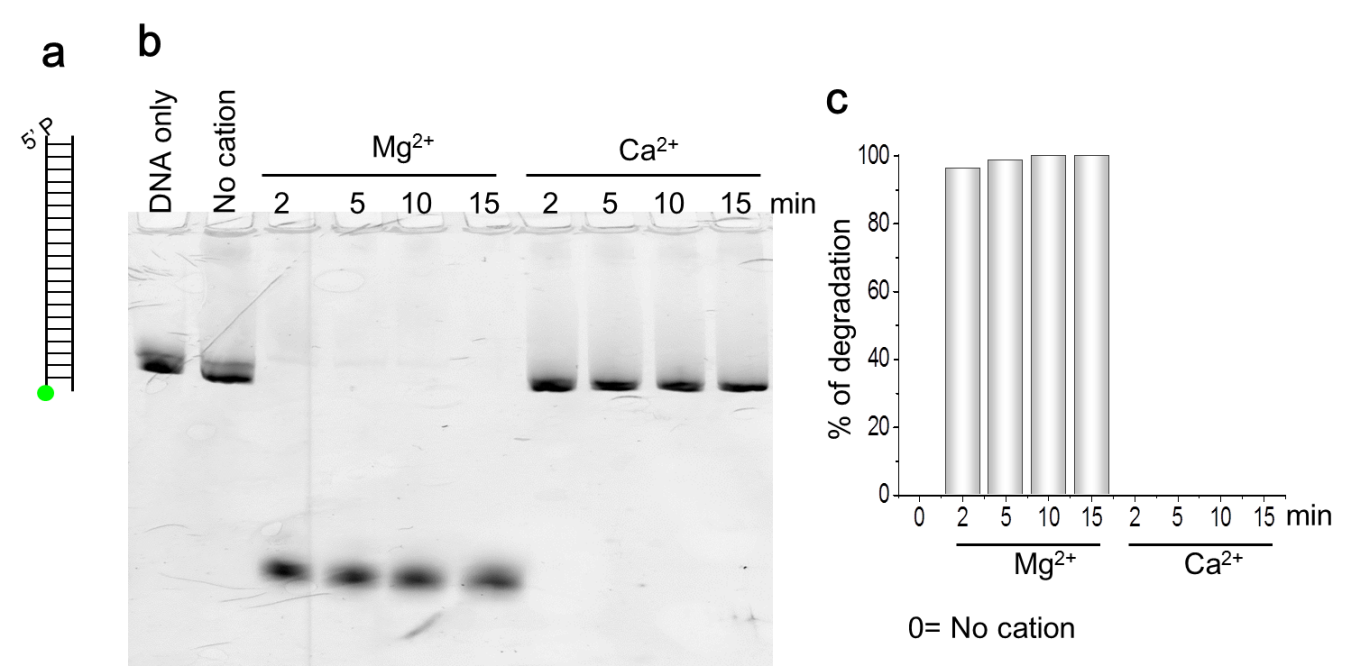
**

**Supplementary Figure 5 |** **Mg^2+^ ions are required as a cofactor to catalyze the nucleotide cleavage reaction, whereas Ca^2+^ ions inhibit the enzymatic activity.** **(a)** DNA substrate used for single-molecule experiments as used for the gel assay. **(b)** Exonuclease assays after treating the DNA with the enzyme (16 nM) for 2, 5, 10, and 15 min. The 5’ strand in the duplex was efficiently degraded in less than 2 min (see the time course of Mg^2+^ in b), whereas it was not degraded at all in the presence of Ca^2+^. **(c)** The percentage of degradation was measured by ImageJ (NIH) from the gel assay in b. The data was in agreement with the known property of λ-exonuclease, performing processive DNA degradation. Otherwise, intermediate bands representing incompletely degraded DNA would have been observed.


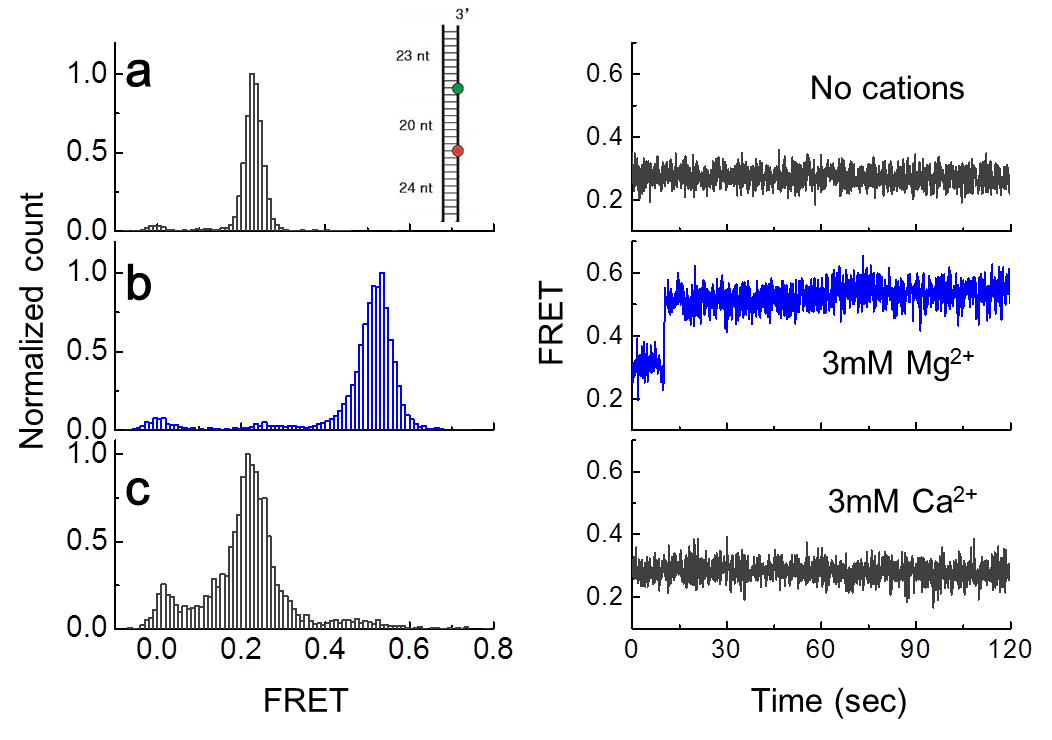


**Supplementary Figure 6 |** **Histograms and corresponding FRET time trajectories as control experiments. (a)** Histogram and a representative trace in the absence of Mg^2+^. **(b)** in the presence of Mg^2+^ at 3 mM. **(c)** in the presence of Ca^2+^ at 3 mM. The single-molecule data was consistent with the gel assay shown in the supplementary Figure 3.


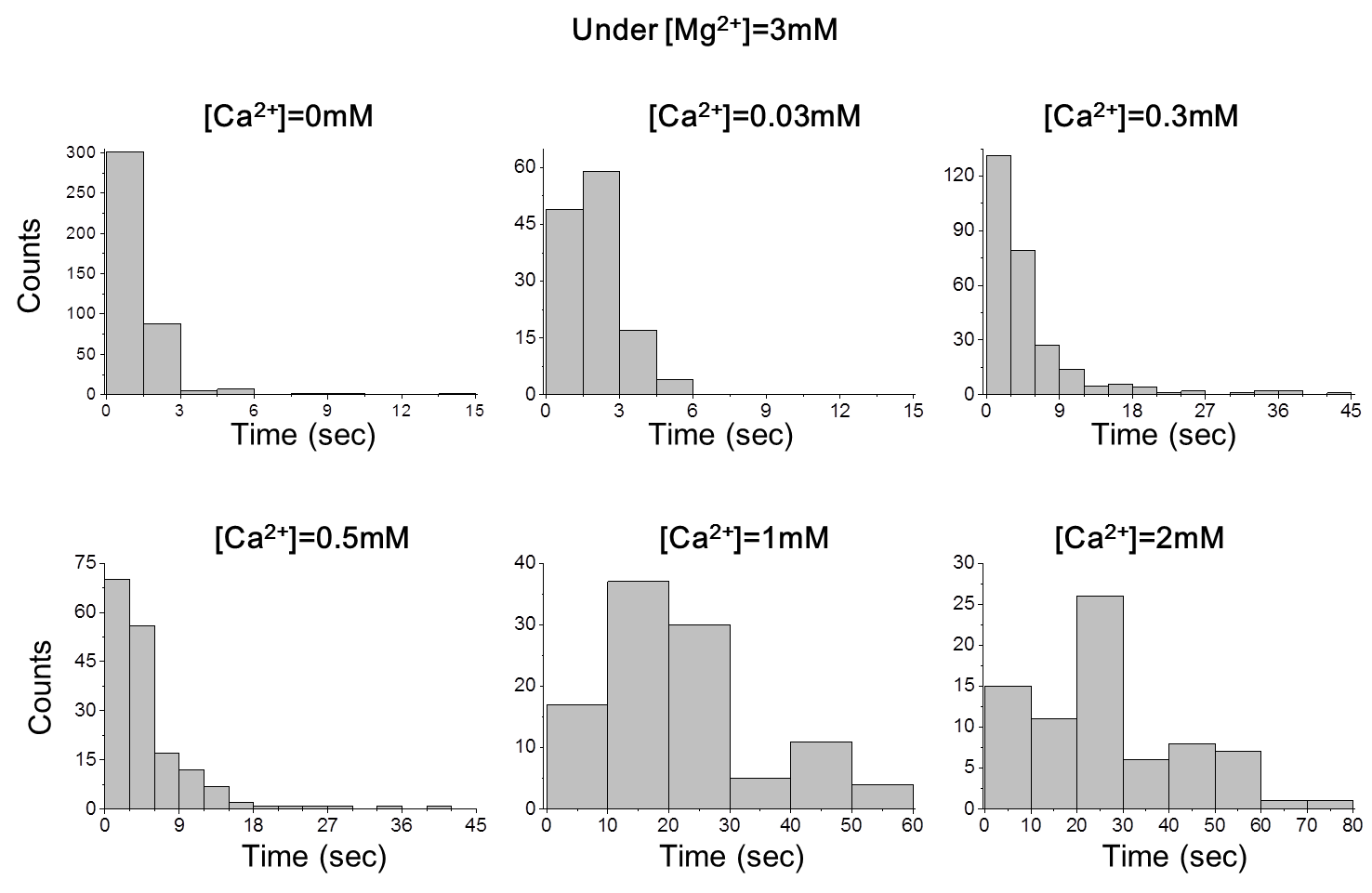


**Supplementary Figure 7 |** Distributions of the pause dwell time as the concentration of Ca^2+^ was increased from 0 to 2 mM at a fixed concentration of 3 mM Mg^2+^.


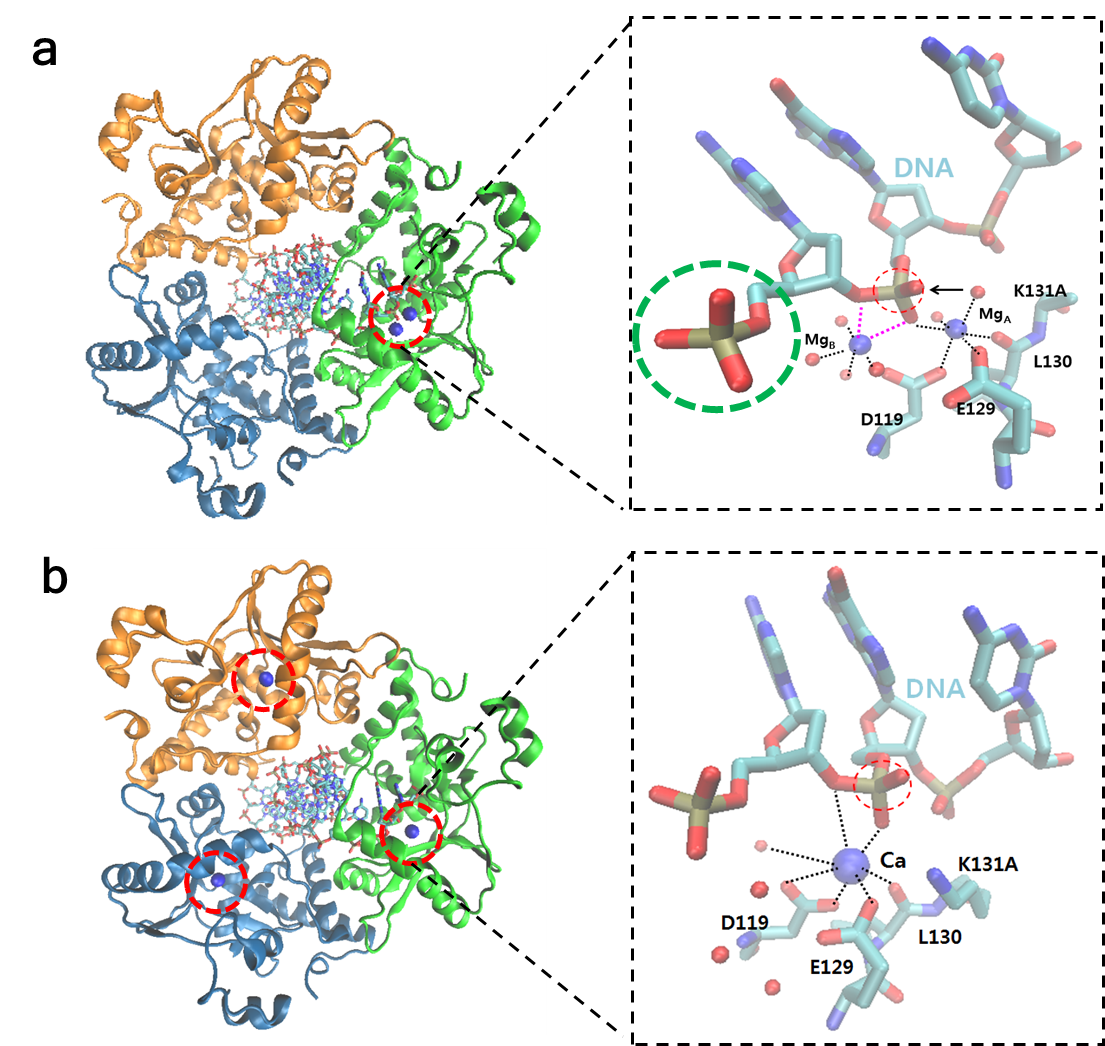


**Supplementary Figure 8 | Coordination around metal ions bound to the active site of λ-exonuclease**

**a**, Mg^2+^-bound structure[^12^](#_ENREF_12) (PDB entry 3SM4). Among the three subunits (orange, green, blue), only the DNA-bound subunit (green) contains the two Mg^2+^ ions. (zoomed-in view) Two metal ions, interacting with active site residues, the scissile phosphate (red circle), and the chelating water molecules (red balls). The black arrow depicts the nucleophilic attack of the hydrolytic water on the scissile phosphate. Black dashed lines illustrate the full octahedral coordination of the metals. **b**, Ca^2+^-bound structure (PDB entry 4WUZ). All three subunits contain Ca^2+^. (zoomed-in view) Ca^2+^ ion coordinating the scissile phosphate, residues, and waters. Note that the hydrolytic water is absent in the Ca^2+^-bound complex. The images were generated by VMD[^13^](#_ENREF_13) (Visual Molecular Dynamics).


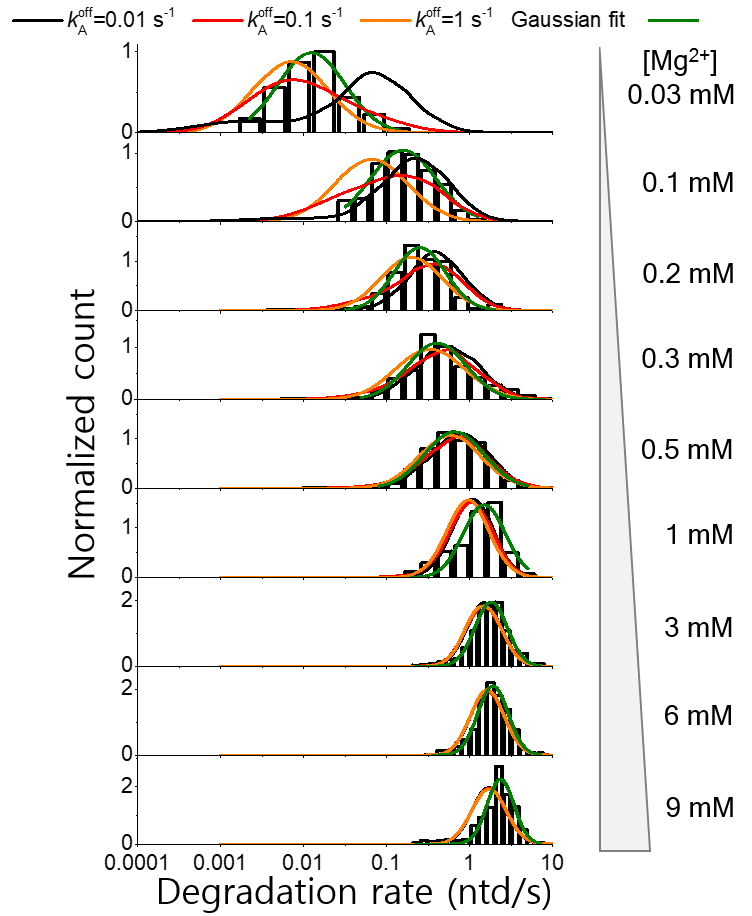


**Supplementary Figure 9 |The degradation velocity histograms.** The bars represent expereimental data, and green solid lines indicate the fit to the Gaussian distribution. Other solid lines represent the theoretical predictions calculated under $k_{A}^{\mathrm{off}}=0.01 s^{-1}$ (black), $k_{A}^{\mathrm{off}}=0.1 s^{-1}$ (red), and $k_{A}^{\mathrm{off}}=1 s^{-1}$ (orange).


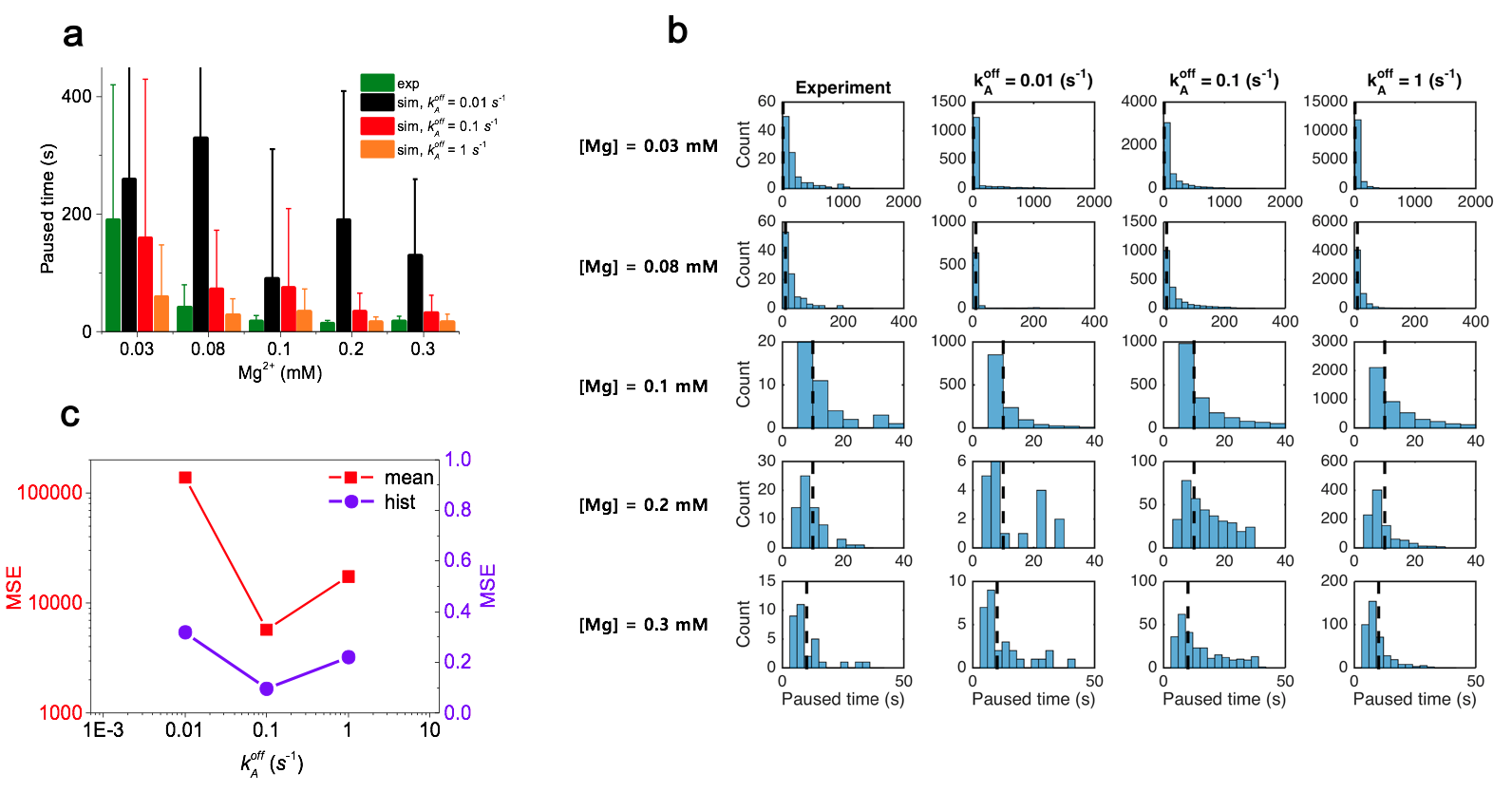


**Supplementary Figure 10 | Paused time analysis.** **(a)** Bar graph of pause time versus Mg^2+^ concentrations. The paused times collected from experiments are coloured in green whereas the paused times generated from simulations under varying $k_{A}^{\mathrm{off}}$are coloured in black ($k_{A}^{\mathrm{off}}=0.01 s^{-1}$), red ($k_{A}^{\mathrm{off}}=0.1 s^{-1}$), and orange ($k_{A}^{\mathrm{off}}=1 s^{-1}$). (**b)** Pause time histograms from the experimental data (first column) and the simulated FRET time trajectories generated under $k_{A}^{\mathrm{off}}=0.01 s^{-1}$ (second column), $0.1 s^{-1}$ (third column), and $1 s^{-1}$ (fourth column). Black dashed lines indicate 10 *s*. (c**)** Mean squared error (MSE) of mean paused time (red) and of paused time histogram (purple) versus $k_{A}^{\mathrm{off}}$. MSEs were calculated by summing the square of the differences between the experimental and simulated results. Large error bars in the experimental data and the simulation result in Supplementary Fig. 10a are due to dynamical heterogeneity (or dynamic disorder) (see **SI** for details).


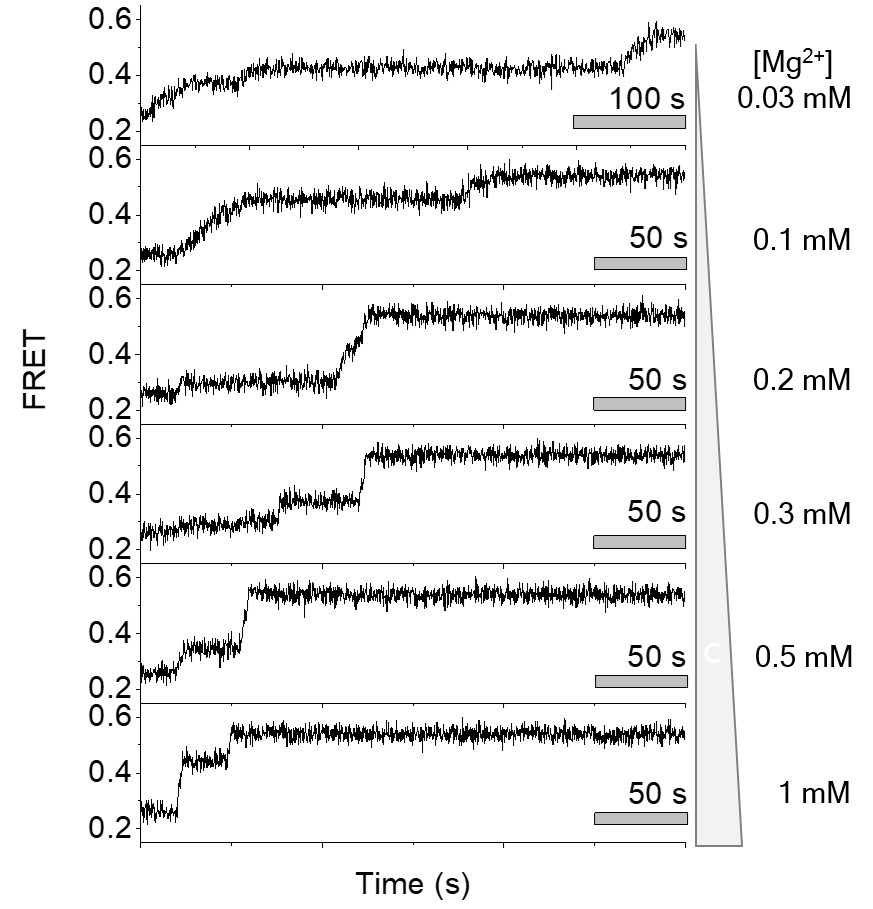


**Supplementary Figure 11 | Representative FRET time trajectories from the simulation.** Traces are generated at $k_{A}^{\mathrm{off}}=0.1 s^{-1}$ with varying [Mg] (from top to bottom, [Mg] = 0.03 mM, 0.1 mM, 0.2 mM, 0.3 mM, 0.5 mM, 1 mM).


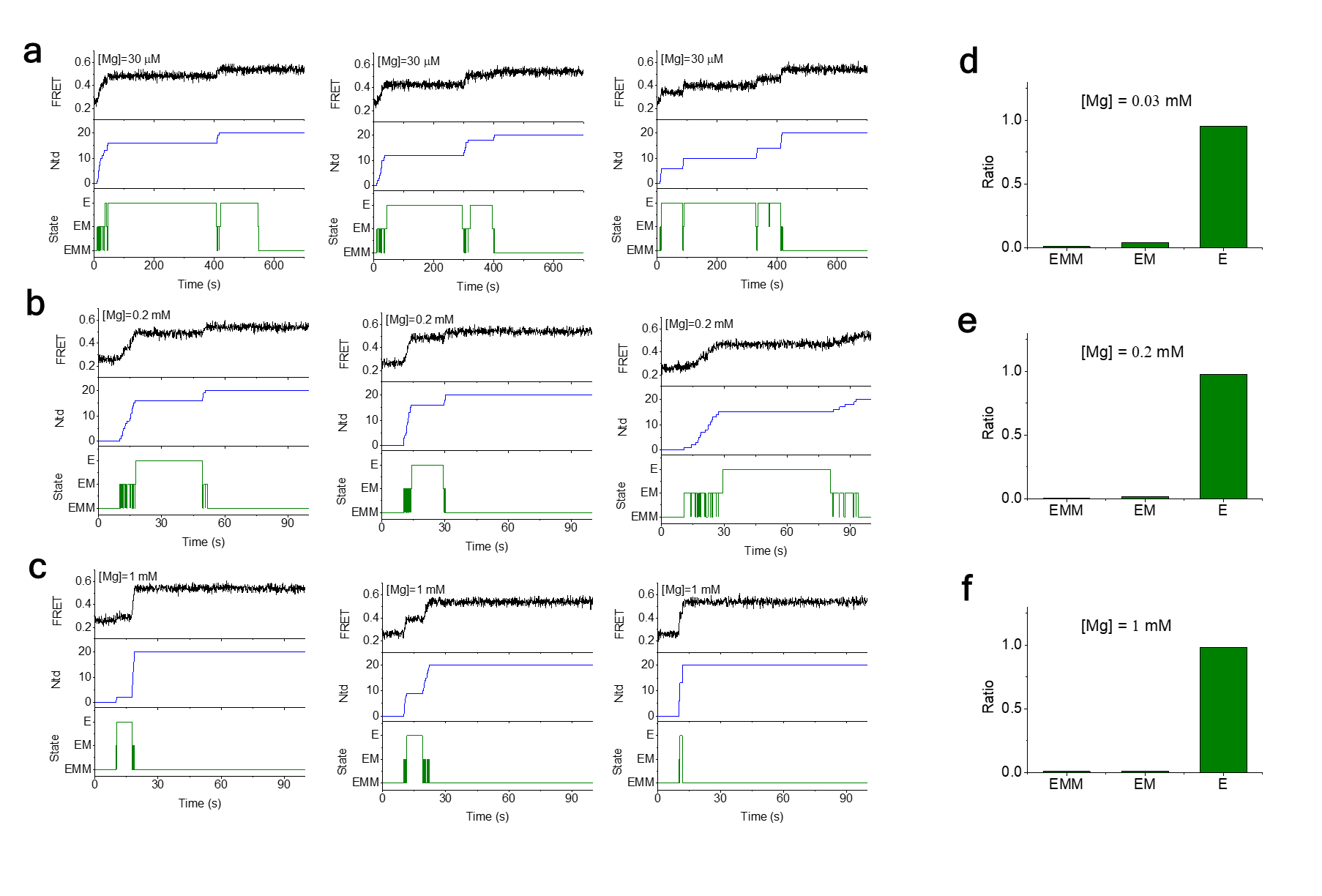


**Supplementary Figure 12 | Representative simulation trajectories and fractions of metal-ion states in the paused state. (a-c**) Representative simulation trajectories under [Mg] = 0.03 mM (a), 0.2 mM (b), and 1 mM (c): a time-FRET trace (black line in the top panel), degradation position of exonuclease along the DNA (the line in middle panel) and its metal ion state (green line in bottom panel). **(d-f)** Fractions of metal ion states in paused state calculated under [Mg] = 0.03 mM (d), 0.2 mM (e), and 1 mM (f).


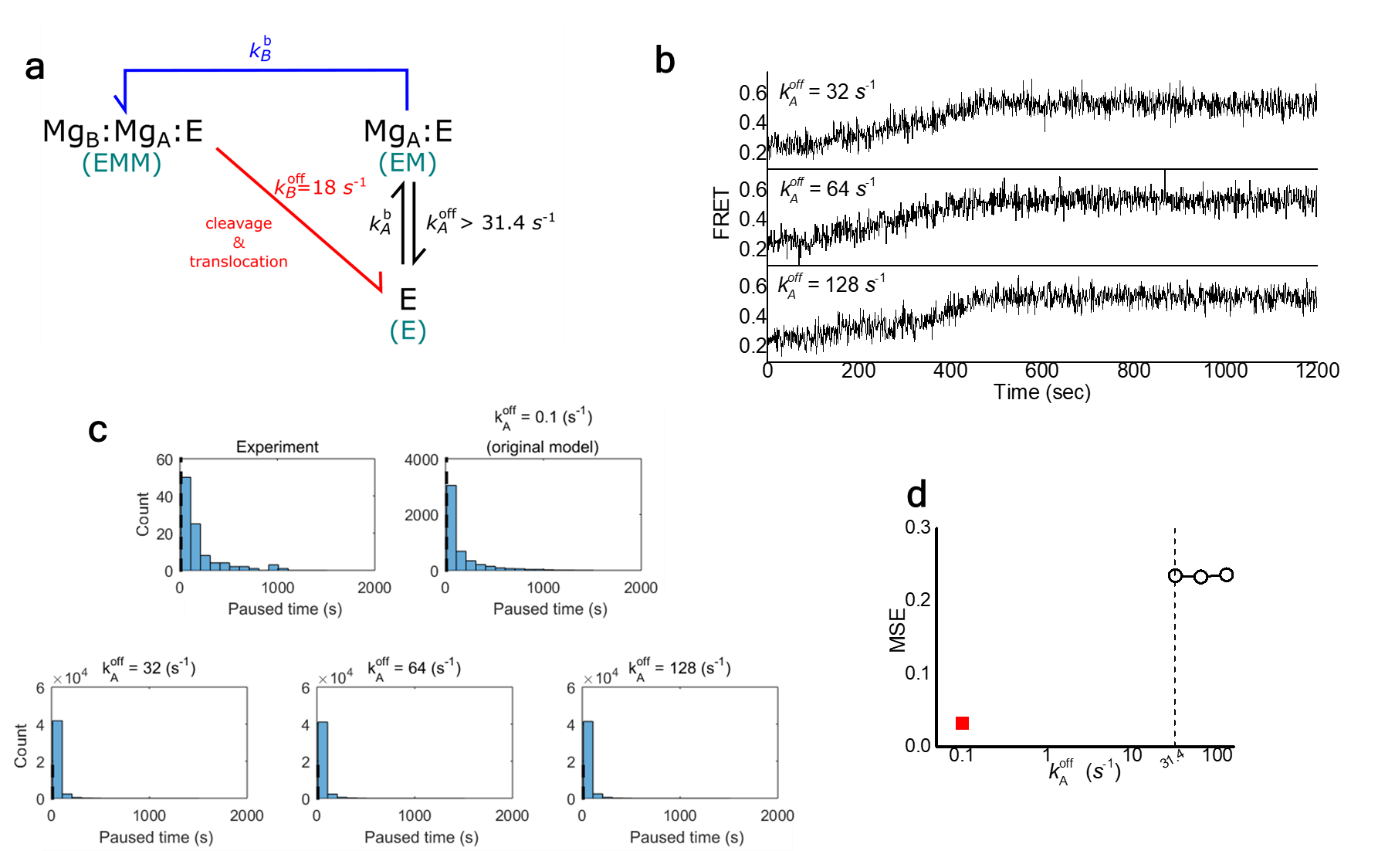


**Supplementary Figure 13 | Kinetic analysis of two-metal dissociation.**

**(a**) Kinetic model-2. Unlike the model shown in Fig. 4a (model-1), both Mg_A_^2+^ and Mg_B_^2+^ ions dissociate upon cleavage and subsequent translocation event. **(b)** Simulated FRET time trajectories at [Mg^2+^]=0.03 mM with varying $k_{A}^{\mathrm{off}} (32, 62,$ and $128 s^{-1}$). **(c)** Comparison of pause-time histograms at [Mg^2+^] = 0.03 mM with varying $k_{A}^{\mathrm{off}}$. The experimental result (upper left panel) and the simulation result of model-1 (upper right panel) (Fig. 4a) are provided in the upper panels. Three panels at the bottom show the simulation results of model-2. **(d)** Mean square error between pause-time histogram of experimental result and simulation results (model-1: red-square, model-2: black circle). Dashed line indicates lower bound of $k_{A}^{\mathrm{off}}$ in model-2, resulting from the fit of the degradation time per nucleotide ($\tau_{1}$) versus Mg^2+^ concentrations in Fig. 4b.


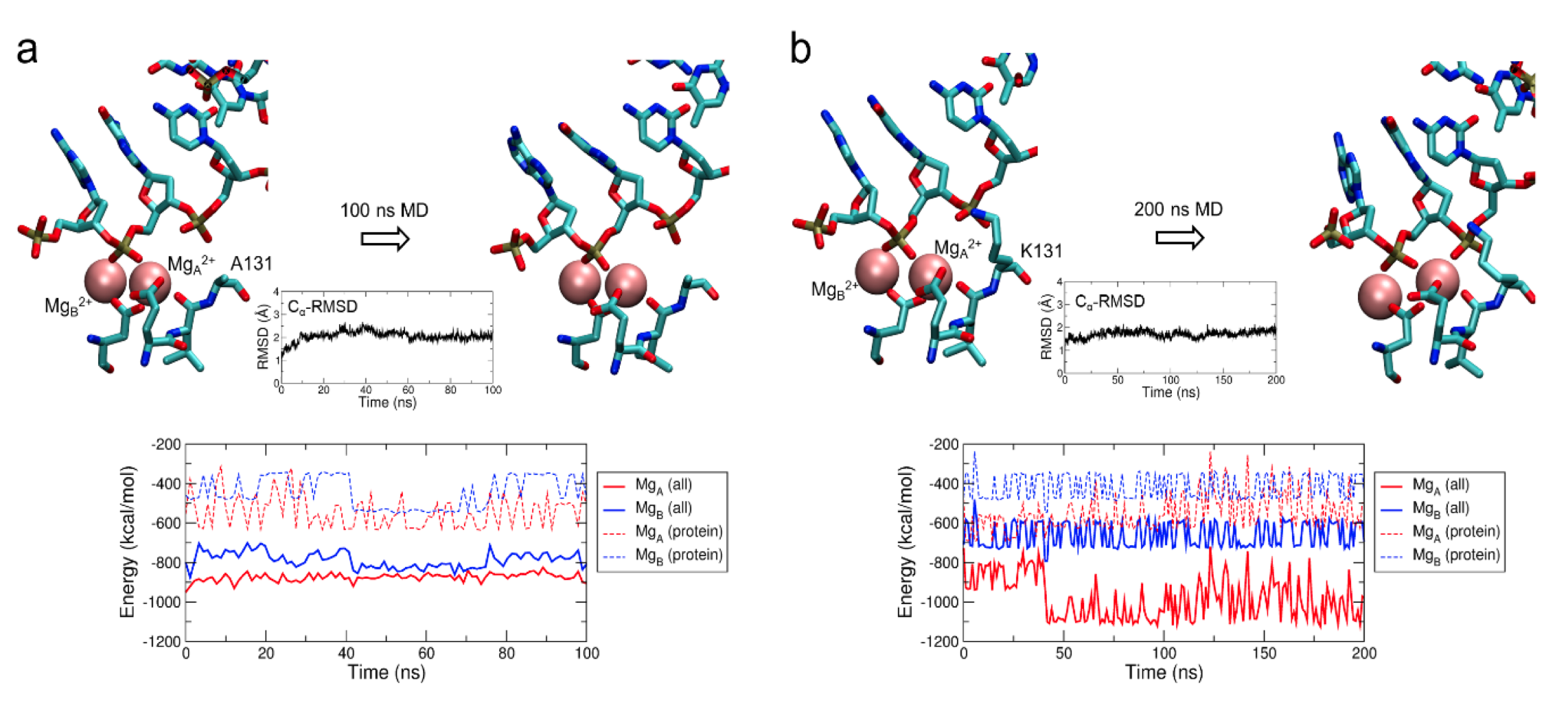


**Supplementary Figure 14 | Comparison of the stabilities of Mg_A_^2+^ (right) and Mg_B_^2+^ using all-atom molecular dynamic (MD) simulation.**

**(a)** The results of MD simulation performed using the crystal structure (PDB ID: 3SM4) whose 131^st^ lysine in the wild-type is mutated to alanine. Magnesium ions are represented as magenta spheres, and nucleic acids and binding residues are shown in the stick representation. Left and right figures depict the snapshots of MD simulation before and after the 100 ns simulations, respectively. Time evolution of C_α_-RMSD is provided in the middle panel. The panel at the bottom show time traces of non-bonded energies of Mg_A_^2+^ (red) and Mg_B_^2+^ (blue). Solid lines (‘all’) represent the sum of interaction energy between the Mg ion and its surrounding protein residues and nucleic acids, whereas the dashed lines indicate the energy from the interaction between the Mg ion and protein only. **(b)** Same MD simulation was repeated for the wild type that retains 131^st^ lysine. After the local rearrangement of the structure, which engenders stable salt-bridge formation between the oxygen atom in the 3^rd^ phosphate group and the K131 side chain, Mg_A_^2+^ ion is more tightly coordinated by the surrounding residues than that in the mutant case in (**a**).

References

1. Aslanidis C, De Jong PJ. Ligation-independent cloning of PCR products (LIC-PCR). *Nucleic Acids Research* **18**, 6069-6074 (1990).

2. *Single-Molecule Techniques: A Laboratory Manual*, 1 edn. Cold Spring Harbor Laboratory Press.

3. Abraham MJ*, et al.* Gromacs: High performance molecular simulations through multi-level parallelism from laptops to supercomputers. *SoftwareX* **1-2**, 19-25 (2015).

4. Berendsen HJC, van der Spoel D, van Drunen R. GROMACS: A message-passing parallel molecular dynamics implementation. *Computer Physics Communications* **91**, 43-56 (1995).

5. Best RB*, et al.* Optimization of the additive CHARMM all-atom protein force field targeting improved sampling of the backbone φ, ψ and side-chain χ 1 and χ 2 Dihedral Angles. *Journal of Chemical Theory and Computation* **8**, 3257-3273 (2012).

6. Hart K, Foloppe N, Baker CM, Denning EJ, Nilsson L, MacKerell Jr AD. Optimization of the CHARMM additive force field for DNA: Improved treatment of the BI/BII conformational equilibrium. *Journal of Chemical Theory and Computation* **8**, 348-362 (2012).

7. Bussi G, Donadio D, Parrinello M. Canonical sampling through velocity rescaling. *Journal of Chemical Physics* **126**, (2007).

8. Parrinello M, Rahman A. Polymorphic transitions in single crystals: A new molecular dynamics method. *Journal of Applied Physics* **52**, 7182-7190 (1981).

9. Darden T, York D, Pedersen L. Particle mesh Ewald: An N·log(N) method for Ewald sums in large systems. *The Journal of Chemical Physics* **98**, 10089-10092 (1993).

10. Lee Y, Thirumalai D, Hyeon C. Ultrasensitivity of Water Exchange Kinetics to the Size of Metal Ion. *Journal of the American Chemical Society* **139**, 12334-12337 (2017).

11. Allnér O, Nilsson L, Villa A. Magnesium ion-water coordination and exchange in biomolecular simulations. *Journal of Chemical Theory and Computation* **8**, 1493-1502 (2012).

12. Zhang J, McCabe KA, Bell CE. Crystal structures of λ exonuclease in complex with DNA suggest an electrostatic ratchet mechanism for processivity. *Proceedings of the National Academy of Sciences of the United States of America* **108**, 11872-11877 (2011).

13. Humphrey W, Dalke A, Schulten K. VMD: Visual molecular dynamics. *Journal of Molecular Graphics* **14**, 33-38 (1996).
